# BSA-Based Stabilization of Prussian Blue-Coated Manganese Ferrite Nanoparticles for MRI

**DOI:** 10.3390/pharmaceutics18101217

**Published:** 2026-09-24

**Authors:** Ágnes Mária Ilosvai, Fatemeh Heydari, László Forgách, Babak Minofar, Noémi Kovács, Krisztián Szigeti, Ferenc Kristály, Lajos Daróczi, Miklós Németh, Tamás Ollár, David Reha, Béla Viskolcz, László Vanyorek

**Affiliations:** 1 Institute of Chemistry, University of Miskolc, Miskolc-Egyetemváros, 3515 Miskolc, Hungary; maria.agnes.ilosvai@uni-miskolc.hu (Á.M.I.); bela.viskolcz@uni-miskolc.hu (B.V.); 2 Higher Education and Industrial Cooperation Centre, University of Miskolc, 3515 Miskolc, Hungary; babak.minofar@uni-miskolc.hu; 3 Department of Biophysics and Radiation Biology, Semmelweis University, 1094 Budapest, Hungary; fatemeh.heydari@stud.semmelweis.hu (F.H.); forgach.laszlo@med.semmelweis-univ.hu (L.F.); noemi.kovacs@hcemm.eu (N.K.); szigeti.krisztian@med.semmelweis-univ.hu (K.S.); 4 In Vivo Imaging Advanced Core Facility, Hungarian Center of Excellence for Molecular Medicine (HCEMM),6728 Szeged, Hungary; 5 Department of Physical Chemistry, Faculty of Chemistry, University of Lodz, Pomorska 163/165, 90-236 Lodz, Poland; 6 Institute of Mineralogy and Geology, University of Miskolc, 3515 Miskolc, Hungary; askkf@uni-miskolc.hu; 7 Department of Solid State Physics, University of Debrecen, P.O. Box 2, 4010 Debrecen, Hungary; lajos.daroczi@science.unideb.hu; 8 Institute for Energy Security and Environmental Safety, HUN-REN Centre for Energy Research, Konkoly-Thege M. 29–33, 1121 Budapest, Hungary; nemeth.miklos@ek.hun-ren.hu (M.N.); ollar.tamas@ek.hun-ren.hu (T.O.); 9 IT4Innovations, VSB─Technical University of Ostrava, 17. Listopadu 2172/15, 708 00 Ostrava, Czech Republic; rehadavid@gmail.com

**Keywords:** magnetic nanoparticles, Prussian blue, MRI, ferrite

## Abstract

**Objectives**: Magnetic nanoparticles are promising candidates for T2-weighted magnetic resonance imaging (MRI), but their physicochemical stability and biological compatibility remain important challenges for biomedical applications. **Methods**: In this study, amine-functionalized manganese ferrite (MnFe_2_O_4_) nanoparticles were coated with Prussian blue (PB) and subsequently formulated with bovine serum albumin (BSA). The resulting formulation was characterized using X-ray diffraction, transmission electron microscopy, X-ray photoelectron spectroscopy, Fourier-transform infrared spectroscopy, magnetic measurements, dynamic light scattering, molecular dynamics simulations, cytocompatibility assays, and MRI relaxometry. **Results**: The BSA-formulated nanoparticles were readily redispersed after lyophilization and remained colloidally stable throughout the 6 h observation period in the tested aqueous media. Molecular dynamics simulations demonstrated persistent close contacts between the MnFe_2_O_4_ surface and BSA residues, supporting stable nanoparticle–protein association. MRI relaxometry showed predominantly T2-weighted contrast behavior, with substantially higher r_2_ than r_1_ relaxivity. In vitro metabolic activity measurements demonstrated higher relative metabolic activity for the PB-coated formulation than for the corresponding uncoated formulation within the BSA-formulated systems. A pilot in vivo MRI experiment performed in a single mouse demonstrated rapid hepatic accumulation, with a detectable reduction in liver signal intensity within 10 min after administration and persistence of the hepatic signal change at the following-day measurement. **Conclusions**: These preliminary findings support further investigation of BSA-formulated PB-coated MnFe_2_O_4_ nanoparticles as a potential T2-weighted MRI contrast platform.

## 1. Introduction

Magnetic resonance imaging (MRI) is an extremely useful diagnostic procedure that is widely used. This diagnostic method can be further improved by using contrast agents. For contrast-enhanced MR imaging, different complexes and other derivatives of gadolinium, as contrast agents, were approved by the United States FDA; these are gadolinium chelate, gadoterate meglumine, gadobenate dimeglumine, gadodiamide, gadoversetamide, and gadofosveset trisodium, with brand names Magnevist, Dotarem, Prohance, Omniscan, Optimark, Gadavist, and Primovist [1]. Application of gadolinium-based contrast agents can have a toxic effect on living organisms due to the unexpected release of free Gd ions [2,3]. In order to avoid the aforementioned toxicity, the Gd-based contrast materials are increasingly being displaced by iron oxide-based magnetic nanoparticles in clinical applications. However, iron oxide-based contrast agents are not without limitations, and their clinical uptake has also been moderated by reports of adverse effects and iron overload. Iron oxide nanoparticles with superparamagnetic properties, namely magnetite and maghemite, are effective T2 contrast agents in MRI diagnostics due to their biocompatibility [4,5]. Furthermore, there are some types of pathologies that cannot be diagnosed sufficiently effectively by Gd-based contrast agents, such as the characterization of focal liver lesions (FLLs). In such cases, the use of superparamagnetic iron oxide (SPIO) contrast agents is appropriate [6]. Several types of superparamagnetic iron oxides have been developed, i.e., Ferumoxides (Feridex^®^), Ferucarbotran (Resovist^®^), Ferumoxtran-10 (Combidex^®^, Sinerem^®^), and citrate-coated superparamagnetic iron oxide particles (VSOP C184), which have been clinically tested as MRI contrast agents [7]. Another SPIO agent, Ferumoxytol (Feraheme^®^), is approved for the treatment of iron deficiency in adult chronic kidney disease patients. However, health safety concerns have also been raised regarding preparations containing superparamagnetic iron oxide. The administration of widely utilized commercial iron-based contrast agents has been associated with diverse adverse effects, ranging from localized pain and hypotension to severe hypersensitivity, anaphylaxis, vasodilation, and paresthesia [5]. The administration of these contrast media may lead to iron overload, which triggers the generation of reactive oxygen species (ROS) through Fenton and Haber–Weiss pathways, ultimately resulting in significant intracellular damage [8,9,10,11,12,13,14,15]. Following reports of the health risks and side effects detailed above, the commercial availability of ferumoxide and ferucarbotran ceased in 2008 and 2009 [5,16,17]. For magnetite and maghemite-based contrast agents, the aim should be to ensure successful imaging in MRI scans with the lowest possible dose, without toxic effect. Surface coating with biocompatible materials, including human serum albumin (HSA) and bovine serum albumin (BSA), has been investigated as an approach to reduce nanoparticle-associated biological effects. BSA and HSA have been investigated as protein-based carriers and stabilizing matrices because of their biocompatibility, aqueous solubility and modifiable surface chemistry [18,19,20,21,22]. Both HSA and BSA can be excellent carriers for anticancer drugs and radiopharmaceuticals [23,24]. There are other ways to reduce toxicity caused by iron overload, such as using complexing agents that cover the surface of magnetic particles, preventing iron from being released. Potassium hexacyanoferrate serves as a promising chelating agent by forming a chemically inert complex upon interaction with iron(III) ions. Prussian blue, also known as iron(II) iron(III) octadecacyanide (Fe^3+^[Fe^3+^Fe^2+^(CN)_6_]_3_), is a biocompatible complex that exhibits significant versatility, finding extensive applications across the fields of nanomedicine and diagnostic imaging [25]. This process has also been successfully applied to maghemite nanoparticles, which were coated with a Fe^3+^[Fe^3+^Fe^2+^(CN)_6_]_3_ layer on their surface [26]. In addition to magnetite and maghemite, a number of magnetic nanoparticles can be produced as oxides of trivalent iron and divalent transition metals, including transition metal ferrites (MFe_2_O_4_, where M: Cu(II), Mn(II), Co(II), Zn(II), etc.), which are spinel-type magnetic metal oxides [27,28,29]. The above-mentioned ferrite nanoparticles are well suited as T2 contrast agents of magnetic resonance imaging [30,31,32]. The spinel-type magnetic nanoparticles (MFe_2_O_4_) can be prepared by several methods; one common process is solvothermal synthesis [33,34,35]. In the solvothermal method, ethylene glycol or polyethylene glycol, next to the surfactants and sodium acetate, is used as the reaction medium. These synthesis methods lead to the formation of nanoparticles with similar morphologies and size [36,37,38].

While superparamagnetic iron oxide nanoparticles (SPIOs) have been widely investigated as $T_{2}$ -weighted magnetic resonance imaging (MRI) contrast agents, conventional formulations continue to face severe clinical and technological limitations. The majority of systems reported in the literature have either not been evaluated in physiological media or suffer from poor long-term colloidal stability.

Our previous study established the synthesis and MRI-related properties of Prussian blue-coated MnFe_2_O_4_ nanoparticles [39]. The present work focuses instead on formulation of an independently prepared batch of these nanoparticles with bovine serum albumin (BSA). Particular attention was given to lyophilization and redispersion, colloidal stability in physiologically relevant media, nanoparticle–BSA interactions, cytocompatibility, and MRI relaxivity.

Additionally, molecular dynamics (MD) simulations were used to investigate the interactions between BSA and the nanoparticle surface and to identify amino acid residues involved in persistent nanoparticle–protein contacts.

## 2. Materials and Methods

### 2.1. Materials

The manganese ferrite nanoparticles were made from the following ingredients: manganese (II) nitrate tetrahydrate—Mn(NO_3_)_2_·4H_2_O (Carl Roth GmbH, Karlsruhe, Germany) and iron(III) nitrate nonahydrate—Fe(NO_3_)_3_·9H_2_O (VWR International, Leuven, Belgium); ethylene glycol—HOCH_2_CH_2_OH, (VWR Int. Ltd., F-94126 Fontenay-sous-Bois, France); monoethanolamine—NH_2_CH_2_CH_2_OH (Merck KGaA, D-64271 Darmstadt, Germany); and sodium acetate—CH_3_COONa (ThermoFisher GmbH, D-76870 Kandel, Germany). Potassium hexacyanoferrate(II) trihydrate—K_4_[Fe(CN)_6_]·3H_2_O and iron(III) chloride (anhydrous)—FeCl_3_ (Sigma Aldrich Ltd., St. Louis, MO 63103, USA) were applied for ferric ferrous cyanide coating of the ferrite nanoparticles. Bovine serum albumin (Sigma Aldrich Ltd., St. Louis, MO 63103, USA) was used as a support and stabilizing material for the magnetic nanoparticles.

### 2.2. Characterization Techniques

Structural and phase characterizations of the magnetic nanoparticles were conducted using a Talos F200X G2 high-resolution transmission electron microscope (HRTEM), equipped with an X-FEG field-emission gun operated between 20 and 200 kV. Microscopic imaging and selected area electron diffraction (SAED) patterns were acquired via a 16-Mpixel Ceta 4k × 4k CMOS SmartCam digital search camera featuring a high-angle annular dark-field (HAADF) detector. Samples were prepared by drop-casting an aqueous dispersion of the nanoparticles onto carbon-coated copper grids (Ted Pella Inc., Redding, CA, USA).

The crystalline phases of the MnFe_2_O_4_ and PB-coated MnFe_2_O_4_ samples were characterized by X-ray diffraction (XRD) using a Bruker Discovery diffractometer. Measurements were carried out using a Cu-K-α radiation source operating at 40 kV and 40 mA. A parallel-beam arrangement was set up via a Göbel mirror, with diffraction patterns recorded using a Vantec detector. Crystalline phases were assigned by comparison against powder diffraction files (PDFs). To determine the mean crystallite domain size of the manganese ferrite phase, profile fitting was performed using TOPAS 4 software; the evaluation relied on the calibrated mean column length approach, utilizing both the full width at half maximum (FWHM) and the Lorentzian peak profile width.

Surface elemental content and oxidation states were evaluated using a Thermo Scientific ESCALAB Xi+ spectrometer, equipped with a monochromatic Al K-α X-ray source (1486.6 eV) set to a spot diameter of 650 µm. Due to the ferromagnetic nature of the specimens, the electron transfer lens was operated in electrostatic mode. Overall composition surveys were obtained by recording wide-range spectra at an analyzer pass energy of 80 eV. For accurate chemical state determination and quantification, high-resolution core-level spectra were gathered at a pass energy of 20 eV for the C 1s, O 1s, Mn 2p, Fe 2p, N 1s, Mn 3s, and Fe 3p regions. Surface charging was neutralized using the system’s integrated automatic dual-charge compensation unit. Calibration of the binding energy scale was conducted by fixing the sp3 C 1s (C–C/C–H) peak position at 284.8 eV. Elemental quantification was calculated under the assumption that the material was homogeneous within the probed XPS volume, with an estimated measurement error within ±10%.

To identify surface functional groups, the manganese ferrite samples were characterized in transmission mode using a Bruker Vertex 70 Fourier-transform infrared (FTIR) spectrometer. Sample preparation involved pressing 15 mg of powder with 250 mg of spectroscopic-grade KBr into pellets.

The magnetic behavior of the ferrite nanoparticles was studied using a custom-built vibrating-sample magnetometer (University of Debrecen, P.O. Box 2, 4010 Debrecen, Hungary) powered by a water-cooled Weiss-type electromagnet. Powder samples with a typical mass of 20 mg were pelletized prior to analysis. Field-dependent magnetization (M vs. H) was recorded at room temperature under applied fields up to 10,000 Oe.

Hydrodynamic diameter and surface charge (zeta potential) were characterized using a Litesizer 500 instrument at 25 °C (Anton Paar, Hamburg, Germany). Dynamic light scattering (DLS) measurements were conducted at 25 °C utilizing a He-Ne laser source operating at a wavelength of 633 nm. Measurements were recorded in automatic mode at three distinct detection angles: 175° (backscatter), 90° (side scatter), and 15° (front scatter). Measurements were carried out using single-use polystyrene cuvettes sourced from Anton Paar (Hamburg, Germany).

Human embryonic kidney (HEK 293T) cells, sourced from ATCC (Manassas, VA, USA), were cultivated at 37 °C and 5% CO_2_ under humidified conditions. The growth medium consisted of Dulbecco’s Modified Eagle’s Medium (DMEM; Lonza) enriched with 10% fetal bovine serum, alongside penicillin (50 U/mL) and streptomycin (50 µg/mL). For toxicology assays, cells were seeded onto black, flat-bottom 96-well plates (Greiner Bio-One, ref. 655076) at a density of 1 × 10^4^ cells per well and allowed to adhere for 24 h. For cytotoxicity testing, BSA-formulated MnFe_2_O_4_ nanoparticles with or without PB coating were applied at ferrite concentrations ranging from 1 mg/mL to 0.01 mg/mL. Ultrapure water and Dulbecco’s Modified Eagle Medium (DMEM) served as positive and negative controls, respectively. Following 24 h of incubation at 37 °C with 5% CO_2_, the culture medium was replaced with a transparent medium for subsequent measurements.

For cytotoxicity testing of the ferrite nanoparticles, alamarBlue^®^ Cell Viability Reagent (Invitrogen) was introduced at a 1:10 dilution factor, followed by a 1 h cell incubation at 37 °C. Cell viability was assessed according to the manufacturer’s instructions (Thermo Fisher Scientific, alamarBlue^®^ Cell Viability Reagent, Cat. No. DAL1025). A Varioskan Lux multimode microplate reader (Thermo Fisher Scientific, Waltham, MA, USA) was employed for fluorescence detection. Fluorescence was measured using Ex/Em = 570/590 nm on the Varioskan LUX multimode microplate reader (Thermo Fisher Scientific), in accordance with the manufacturer’s recommended peak excitation/emission settings for monochromator-equipped instruments (alamarBlue Product Information Sheet, Thermo Fisher Scientific, Cat. No. DAL1025).

Raw fluorescence signals were recorded as relative fluorescence units (RFU) and subsequently normalized to the untreated and dead-cell controls to obtain relative metabolic activity (%). Using this assay, we evaluated the effect of BSA-formulated MnFe_2_O_4_ nanoparticles with and without PB coating on the metabolic activity of HEK 293T cells. Relative viability was calculated by normalizing fluorescence signals to dead (0%) and untreated (100%) controls, and data are reported as mean ± SD from triplicate wells.

Relative viability (%) was derived via the following equation (Equation (1)):

(1) $$\mathrm{Relative}\;\mathrm{viability}\;(\%)=\frac{RFU_{\mathrm{sample}}-RFU_{\mathrm{dead}}}{RFU_{\mathrm{untreated}}-RFU_{\mathrm{dead}}}\times 100$$

where:

• $RFU_{\mathrm{sample}}$ = fluorescence signal of the nanoparticle-treated well;
• $RFU_{\mathrm{dead}}$ = average fluorescence of dead control wells (0% viability);
• $RFU_{\mathrm{untreated}}$ = average fluorescence of untreated control wells (100% viability).

Data are reported as mean ± SD of triplicate wells.

Scans were acquired using a 3 T nanoScan^®^ PET/MRI scanner (Mediso Ltd., Budapest, Hungary). In vitro scans were conducted on four different manganese ferrite concentrations (0.002, 0.005, 0.01, 0.02 mg/mL) in 2 mL Eppendorf tubes. The geometrical parameters of the scans were the same: one 4 mm thick coronal slice, and images were acquired with a 50 mm field of view (FOV) and an in-plane spatial resolution of 0.36 mm. T_1_ relaxation times (longitudinal relaxation time) were determined using a multi-inversion recovery (multi-IR) spin-echo sequence (TR = 5.2 s, TE = 5.8 ms) with inversion times (TI) of 0.1, 0.4, 0.6, 0.8, 1, 1.3 and 2 s. T_2_ mapping was performed via a multi-echo spin-echo sequence (TR = 3856 ms), utilizing an initial echo time of 5.5 ms followed by 32 subsequent echoes at 5.55 ms intervals. For T_2_* calculations, a 2D multi-echo gradient-recalled echo (GRE) sequence was employed (TR = 350 ms, minimum TE = 1.71 ms), consisting of 32 echoes with 1.91 ms intervals and a total acquisition time of 35 min.

To execute in vivo MRI scans, a female BALB/c mouse was anesthetized using isoflurane (Arrane^®^, Baxter, Newbury, UK). Sedation was induced at a 5% concentration and subsequently held between 1.5% and 2% throughout the imaging procedure to preserve a suitable anesthetic depth. The fat-saturated T2-weighted fast spin echo (FSE) scans were collected at five different time points (pre-injection, 10 min, 25 min, 40 min, and 1-day p.i.). An 80 × 50 mm FOV on 20 coronal slices was acquired, with a matrix size of 256 × 200, slice thickness of 1 mm, 8 averages, and TR/TE of 4800 ms/40 ms. The scan time was 8 min.

Animals were maintained under controlled ambient conditions (22 ± 2 °C) with a standard 12 h light/dark schedule, while standard chow and water were provided ad libitum. Animal health and behavior were monitored daily during the experimental period. No animals showed signs of severe distress or reached predefined clinical endpoints (e.g., >20% weight loss, labored breathing, or prolonged lethargy) during the study. At the end of the 24 h imaging period, animals were euthanized by overdose of Euthasol (400 mg mL^−1^ sodium pentobarbital, dose: 150 mg/kg i.p.). Animal handling, MRI measurements, and animal experiments were performed by Dr. László Forgách, Dr. Noémi Kovács, and Fatemeh Heydari at the Department of Biophysics and Radiation Biology, Semmelweis University, under the supervision of laboratory head Dr. Krisztián Szigeti.

All procedures involving animals were conducted in accordance with the ARRIVE guidelines and the guidelines set by the European Communities Council Directive (86/609 EEC). The study was approved by the Animal Care and Use Committee of Semmelweis University (approval number PE/EA/01319-6/2023).

### 2.3. Synthesis of the Amine Functionalized Ferrite Nanoparticles

During the synthesis of the NH_2_-functionalized MnFe_2_O_4_ magnetic nanoparticles, firstly, the iron (2 mmol) and manganese (1 mmol) precursors were dissolved in 50 mL ethylene glycol. Another solution was made from sodium acetate (15 mmol) in 100 mL ethylene glycol which was heated to 198 °C under reflux and continuous stirring. The two solutions were mixed, after ethanol amine (35 mL) was added to the heated, metal precursor-containing solution, which was boiled for 12 h next to continuous agitation and reflux. The nanoparticles were separated from the solution using a magnet. After being resuspended in distilled water, the solid residue was repeatedly rinsed with distilled water to ensure thorough washing. The formation of manganese ferrite is promoted by ammonia released from the transformation of ethanolamine, because the presence of manganese(II) ions reduces the thermal decomposition temperature of the ethanolamine [40]. Additionally, Rochell demonstrated that iron(III) species catalyze ethanolamine oxidation. Mechanistically, this pathway generates an aminium radical intermediate that undergoes deprotonation to yield an imine radical, which subsequently decomposes, by way of an imine species into aldehyde (hydroxyacetaldehyde) and ammonia [41]. The alkaline conditions lead to the formation of iron(III) hydroxide and manganese(II) hydroxide, which can be transformed into MnFe_2_O_4_ through dehydration (Equations (2)–(6)):

(2) $${NH}_{2}{CH}_{2}{CH}_{2}OH\overset{{Fe}^{3+}{and-H}^{+}}{\to }HN{=CH-CH}_{2}-OH+H_{2}O\overset{{Fe}^{3+}}{\to }HO{CH}_{2}CHO+{NH}_{3}$$

(3) $${NH}_{3}+H_{2}O\to {NH}_{4}^{+}+{OH}^{-}$$

(4) $${Fe}^{3+}+3{OH}^{-}\to {Fe(OH)}_{3}$$

(5) $${Mn}^{2+}+{2OH}^{-}\to {Mn(OH)}_{2}$$

(6) $${Mn(OH)}_{2}+{2Fe(OH)}_{3}\to {MnFe}_{2}O_{4}+{4H}_{2}O$$

### 2.4. Deposition of PB Layer on Surface of the NH_2_-Functionalized Manganese Ferrite

Amine functionalized ferrite (in aqueous colloid of 100 mg ferrite) was added to a 50 mL aqueous solution of potassium ferricyanide, K_4_[Fe(CN)_6_] (0.1 millimole), and 10 mL solution of 0.1 millimole of iron(III) chloride was incorporated into the ferrite colloid under continuous ultrasonic treatment. Following these preparatory stages, a Prussian blue (PB) shell—specifically ferric ferrous cyanide, Fe^3+^[Fe^3+^Fe^2+^(CN)_6_]_3_—was formed on the outer shell of the manganese ferrite nanoparticles via the global reaction outlined below (Equation (7)).

(7) $$4\;{FeCl}_{3}+{3\;K}_{4}[Fe\left({CN)}_{6}\right]={{Fe}_{4}[Fe\left({CN)}_{6}\right]}_{3}+12\;KCl$$

After the washing of the MnFe_2_O_4_ particles, these were redispersed in a 10 mL solution of 900 mg BSA using an ultrasonic bath. The water from the BSA containing dispersion of ferrite nanoparticles was evaporated with freeze drying.

### 2.5. Computational Details for the Molecular Dynamics Simulation Model

The molecular dynamics (MD) simulation model was constructed based on the experimentally resolved crystal structure of bovine serum albumin (BSA) (PDB ID: 3V03), together with three manganese ferrite (MnFe_2_O_4_) nanoparticles (Figure 1A). Before system assembly, the protein structure was carefully inspected to ensure structural integrity and suitability for simulation. Each magnetic nanoparticle was modeled as a spherical particle with a radius of 25 Å (Figure 1B), representing a simplified yet physically meaningful approximation of the nanoparticle geometry. The atomic structure of the nanoparticles was generated using the pymatgen library [42] and subsequently refined and visualized with the Maestro program [43]. The composite BSA–MnFe_2_O_4_ system was assembled by manually positioning three nanoparticles in proximity to the protein surface at selected regions of interest. This initial configuration was designed to enable potential protein–nanoparticle interactions. Following placement, the entire system geometry was optimized to relieve steric clashes and to refine the interfacial contacts between the protein and the nanoparticles, ensuring a physically realistic starting structure for subsequent MD simulations. The MD simulations were performed using GROMACS 2020 [44,45,46] with the AMBER99SB force field. Standard force field parameters were applied to the protein. The parameters for the MnFe_2_O_4_ nanoparticle were derived from a simplified, smaller structural model (Figure 1C) following the standard AMBER parameterization protocol [47]. Atomic partial charges were calculated using the RESP method [48] with the total charge of the MnFe_2_O_4_ model constrained to zero. Atom types for the small MnFe_2_O_4_ model were assigned based on the local chemical environment of each atom. In total, one atom type was defined for Ni atoms, three atom types for Fe atoms, and five atom types for O atoms. These atom types were subsequently transferred to all corresponding atoms in the larger MnFe_2_O_4_ nanoparticle model (spherical particles with a radius of 25 Å) used in the simulations.

MD simulations were performed at 300 K in a cubic simulation box with approximate dimensions of 18.1 × 18.1 × 18.1 nm^3^. The system was solvated with 180,143 TIP3P water molecules, and 20 Na^+^ counterions were added to neutralize the net negative charge of the protein. Periodic boundary conditions were applied in all three XYZ directions. A time step of 2 fs was used, and bond lengths involving hydrogen atoms were constrained using the LINCS algorithm. Production simulations of 100 ns were carried out for both the isolated protein and the protein in complex with three MnFe_2_O_4_ nanoparticles. All simulations were conducted in the NVT ensemble using a Berendsen thermostat for temperature control. Trajectory coordinates were saved every 10 ps for subsequent analysis.

To ensure the feasibility of the MD calculations, we carried them out using a simplified manganese ferrite nanoparticle surface, rather than a particle with a multi-layered PB coating. Although the MD simulation we used does not account for the specific spatial geometry of the cyanide (C≡N) ligands, the model nevertheless accurately reflects the overall electrostatic profile, localized partial charges and hydrogen-bonding sites of the PB-coated surface. The model therefore provides a simplified representation of the electrostatic and hydrogen-bonding interactions that may contribute to association between BSA and the nanoparticle surface.

## 3. Results and Discussion

### 3.1. Characterization of Manganese Ferrite Nanoparticles Synthesized via the Polyol Method

In the diffractograms of the ferrite sample, reflections were found at 2θ values of 18.1°, 29.9°, 35.3°, 42.5°, 52.7°, 56.3°, and 61.7°, which correspond to (111), (220), (311), (400), (422), (511), and (440) Miller indexed crystal lattices of the MnFe_2_O_4_ (PDF 74–2403) phase (Figure 2). No other metal oxide phases were found in the sample.

Based on the TEM images, the MnFe_2_O_4_ nanoparticles formed spherical clusters with an overall diameter of 55 ± 11 nm. These clusters consisted of individual ferrite nanoparticles with sizes of approximately 4–10 nm. (Figure 3A,B). These nanospheres were composed of individual ferrite nanoparticles, and their sizes were between 4 and 10 nm (Figure 3C). The average crystallite size was determined to be 5 ± 2 nm, calculated from the full width at half maximum (FWHM) of the XRD peaks using the Scherrer equation (6 ± 2 nm by mean column length calibrated analysis; 5 ± 2 nm by Scherrer FWHM). The average crystallite size was determined to be 5 ± 2 nm by the Scherrer equation (6 ± 2 nm by mean column length calibrated analysis; 5 ± 2 nm by Scherrer FWHM). SAED analysis identified diffraction rings characteristic of the ferrite structure (Figure 3D). The calculated d-spacing values were correlated with X-ray databases, specifically the powder diffraction file of the manganese ferrite (PDF 74-2403). The results of the SAED are matched with the X-ray diffractograms of MnFe_2_O_4_; reflections were found at 2θ values of 18.2° (111), 29.9° (220), 35.2° (311), 42.4° (400), 52.7° (422), 56.4° (511) and 61.6° (440) (Figure 2). The diameter of the manganese ferrite clusters was 45 ± 13 nm (Figure 3A,B). The size of the nanoparticles that form the spherical clusters (based on XRD measurement) was 6 ± 2 nm; these nano-crystallites are shown on the HRTEM picture (Figure 3C). The SAED image obtained for MnFe_2_O_4_ shows reflections that confirm a spinel structure (Figure 3D).

VSM (vibrating-sample magnetometer) measurements were performed to examine the magnetic properties of the samples (Figure 4). The magnetization curve of the MnFe_2_O_4_ sample was measured at 303 K. The Ms (saturation magnetization) of the MnFe_2_O_4_ nanoparticles was 44.0 emu/g next to the 6.00 kOe magnetic field. Moreover, a hysteresis loop is located on its magnetization curves, whereas the Mr value was 3.7 emu/g and the coercivity (Hc) was 52.0 Oe; these values indicate ferromagnetic behavior (Figure 4). The measured Ms value of these manganese-ferrite nanoparticles is smaller than the Ms value of the bulk phase MnFe_2_O_4_ (82 emu/g) [49]. The average size of the nanoparticles we produced was 55 ± 11 nm; owing to this small particle size, the finite-size effect becomes more pronounced, resulting in lower Ms values for the nanoparticles compared to the bulk phase [50]. The specific magnetization ranges of the reported MnFe_2_O_4_ nanoparticles, from ~16 emu/g to ~78 emu/g, are associated with the particle size and crystal structure [51,52,53,54,55].

### 3.2. Characterization of the Prussian Blue-Coated Manganese Ferrite Nanoparticles

On the high-angle annular dark-field (HAADF) pictures of the Prussian blue (ferric ferrous cyanide)-coated manganese ferrite (MnFe_2_O_4_-PB), nanoparticles appear in sharp contrast (Figure 5). The elemental maps show that the positions of iron, manganese and oxygen elements are the same in the detected area in the case of MnFe_2_O_4_ (Figure 5). This suggests that iron and manganese are present as ferrite in the sample, with no signs of iron and manganese separately on the elemental map. This indicates that ferric ferrous cyanide (Prussian blue) is not present as separate particles from the ferrite nanoparticles but instead is present on their surface.

The FTIR technique was used to identify the functional groups formed on the surface of manganese ferrite nanoparticles (Figure 6). The FTIR spectra of ferrite samples treated and untreated with ferric ferrous cyanide show two characteristic bands that contribute to the metal–oxygen bond vibrations in spinel structures in the range of 400–750 cm^−1^ for tetrahedral and for octahedral complexes. The bands between 800 and 950 cm^−1^ belong to the deformation vibration of the -CH_2_ groups due to the presence of adsorbed ethanolamine and ethylene glycol molecules after the synthesis of the ferrites. The spectra also show the stretching vibrations of the carbon–oxygen bond around 1020 cm^−1^, which originate from the ethylene glycol on the surface of the ferrite particles. The ethylene glycol also contributes to the βOH and νOH vibrations, which show absorption between 1380 and 1500 cm^−1^; moreover, a broad band of the stretching vibration of the hydroxyl group is also seen in the range 3000–3750. Bending and stretching vibration bands indicating the presence of amine functional groups are visible around 1630 cm^−1^ and 3320 cm^−1^. The C=C stretching vibrational band of adsorbed organic molecules was identified around 1580 cm^−1^. The peak around 1660 cm^−1^ is due to the adsorbed water molecules. The adsorbed organic molecules (ethylene glycol) resulted in low-intensity bands between 2800 and 2930 cm^−1^; these are the symmetric and asymmetric stretching vibrational modes of the aliphatic and aromatic C-H bonds, respectively. The presence of surface hydroxyl and amine functional groups on the magnetic nanoparticles enhances their dispersibility within polar media, particularly aqueous solutions. In the case of the sample containing ferric ferrous cyanide (MnFe_2_O_4_-PB), the characteristic vibrational band of the CN bonds in the Fe^2+^-CN-Fe^3+^ fragment appears between 2060 and 2080 cm^−1^ [56,57,58].

Potassium hexacyanoferrate is an effective complexing agent, the use of which may be able to change the composition of the spinel phase. To clarify this, X-ray diffraction studies were performed on a manganese ferrite sample treated with ferric ferrous cyanide. The diffractogram identified reflections that are characteristic of spinel phases of MnFe_2_O_4_; these were located at 2θ values of 18.2° (111), 29.7° (220), 34.9° (311), 42.7° (400), 52.6° (422), 56.4° (511) and 61.7° (440) (PDF 74–2403). (Figure 7). Thanks to the formation of the Prussian blue, additional peaks appeared at 2θ values of 17.6° (200) and 24.9° (220), which belong to the ferric ferrous cyanide (PDF 73-0687). However, in addition to the Prussian blue phase, magnetite was also detected in the sample, which shows characteristic reflections at 2θ values of 18.5° (111), 30.1° (220), 35.5° (311), 43.1° (400), 53.4° (422), 57.1° (511) and 62.5° (440) in the diffractogram (PDF 19-629).

The quantity of magnetite synthesized in conjunction with manganese ferrite was determined to be 34.2 wt%, and the amount of Prussian blue was 6.8 wt% in the MnFe_2_O_4_-PB sample. A redox process takes place between the ${\mathrm{Fe}}^{3+}$ ions on the surface of the manganese ferrite and the [Fe(CN)_6_]^4−^ ions adsorbed onto the surface. Hexacyanoferrate(II) ions readily donate an electron and oxidizes to [Fe(CN)_6_]^3−^ (hexacyanoferrate(III) ions) [59]. This electron is accepted by the surface Fe^3+^ ion of the manganese ferrite, thereby reducing it to an Fe^2+^ ion. The resulting Fe^2+^ ions differ in charge and size from the lattice Fe^3+^, allowing them to detach from the lattice into the solution significantly more easily.

Based on the above, K_4_[Fe(CN)_6_] can act not only as a passive coating, but also as an active reagent; it can reduce surface iron(III) ions to iron(II) ions while simultaneously facilitating their leaching from the manganese ferrite lattice. Based on the above, the presence of potassium hexacyanoferrate and the sonochemical reaction environment promotes the formation and presence of iron(II) ions in the system. Upon adding iron(III) chloride solution to this mixture, the freshly introduced trivalent iron ions react with the divalent iron ions. Driven by the cavitational energy, this stoichiometric combination of iron ions in a 1:2 ratio initiates the precipitation and crystallization of magnetite onto the surface of the existing manganese ferrite nanoparticles. The elemental maps confirm that the formed magnetite is not present as separate particles, but rather forms the spherical nanoparticles together with the manganese ferrite crystallites. The elemental mapping of the Prussian blue-coated manganese ferrite nanoparticles shows no domains where iron is present separately from manganese (Figure 5).

The presence of Prussian blue was confirmed in the sample by XPS measurement. The XPS spectra of the manganese ferrite sample show the manganese band, as well as identifiable peaks for iron, oxygen, carbon, and nitrogen, the latter of which may originate from amine functional groups and C≡N bonds, indicating the presence of Prussian blue (Figure 8A). Potassium is also present in very small quantities in the samples, originating from potassium hexacyanoferrate.

The high-resolution Mn 2p spectrum exhibits two distinct peaks at binding energies of 641.2 eV and 653.2 eV, corresponding to the Mn 2p_3/2_ and Mn 2p_1/2_ states, respectively, confirming the presence of divalent Mn(II) ions in the manganese ferrite (Figure 8B) [60,61,62,63,64].

The Fe 2p regions of the spectra for both the pristine MnFe_2_O_4_ and the PB-functionalized composite exhibit doublet features, specifically the Fe 2p_1/2_ and Fe 2p_3/2_ signals located at 724.6 and 711 eV binding energy values (Figure 8C). These peaks, along with the satellites (at 733.2 and 719.1 eV), indicate that Fe is present in the form of trivalent iron (Fe^3+^) [62]. The deconvolution of the Fe 2p_3/2_ band reveals a peak characteristic of divalent iron (Fe^2+^) at a binding energy of 708.5 eV, which is characteristic of Prussian blue (Fe^3+^[Fe^3+^Fe^2+^(CN)_6_]_3_) (Figure 8D).

The identification of divalent manganese (Mn^2+^) and trivalent iron (Fe^3+^) ions validates the chemical composition of the MnFe_2_O_4_ spinel phases. This finding is additionally supported by the untreated, Prussian blue-free specimen, which exhibited an iron-to-manganese proportion of 2.0, a value matching the expected stoichiometry of the spinel ferrite phase (Table 1). The phase purity of the manganese ferrite spinel was verified by XRD, which showed no evidence of additional metal oxides (Figure 2).

Elemental analysis of the PB-coated samples revealed an iron:transitional metal ratio of 2.6 for MnFe_2_O_4_-PB (15.3% Fe and 5.8% Mn). The excess of iron observed in the Prussian blue-containing ferrite sample can be attributed to the presence of the Prussian blue coating (Fe^3+^[Fe^3+^Fe^2+^(CN)_6_]_3_) and the Fe_3_O_4_ phase, which was also confirmed by XRD measurement (Figure 7).

Potassium was also detected at 0.7 (n/n)% in the MnFe_2_O_4_-PB sample, originating from the K_4_[Fe(CN)_6_] precursor. Furthermore, a substantial increase in nitrogen content from 0.8 (n/n)% to 4.2 (n/n)% for the manganese ferrite confirms the successful incorporation of the nitrogen-rich Prussian blue layer (Table 1).

In the Prussian blue-free ferrite, the lower nitrogen content (0.8 (n/n)%) is attributed to the C-N bonds within the amine functional groups (Table 1). This organic amine-derived C-N peak is located at 400 eV in the N1s spectra (Figure 8A,D). Conversely, the MnFe_2_O_4_- PB sample exhibits a high-intensity band at 397.7 eV, leading to an increased nitrogen concentration (4.2 (n/n)%) due to the C≡N bonds of the Fe^3+^[Fe^3+^Fe^2+^(CN)_6_]_3_ coating (Figure 9A).

The C 1s spectra of the PB-coated MnFe_2_O_4_ samples exhibit distinct peaks at 284.8 eV, 286.1 eV, 287.6 eV, and 288.6 eV. These binding energy values are characteristic of C-C, C≡N, C-OH, C-O-C, C=O, and O-C=O bonds (Figure 9B), which originate from the Prussian blue layer, adventitious carbon, and other adsorbed organic species [63]. The K 2p_3/2_ and K 2p_1/2_ signals observed at 293.2 eV and 295.9 eV, respectively, confirm the presence of residual potassium hexacyanoferrate [64]. Adsorbed organic species from the synthesis, specifically ethylene glycol and ethanolamine, along with adventitious carbon, account for the carbon levels measured in the MnFe_2_O_4_ (22.0 (n/n)%).

The formation of the Prussian blue shell Fe^3+^[Fe^3+^Fe^2+^(CN)_6_]_3_ accounts for the increased carbon content observed in the modified samples (26.5 (n/n)% primarily due to the presence of cyanide (C≡N) ligands (Table 1).

Deconvolution of the O 1s bands revealed specific signals corresponding to lattice oxides at 530.1 eV, along with lattice-bound adsorbed water at 532.5 eV. Additionally, a peak at 531.5 eV was identified, which is attributed to the hydroxyl groups of adsorbed organic species, such as ethylene glycol (Figure 9C) [63]. Functional groups (C-OH, C-O-C, C=O and O-C=O) contribute 6.3% of the total oxygen concentration in the manganese ferrite sample treated with Prussian blue. The largest amount of oxygen is present in the samples in the form of metal oxides and adsorbed water (Table 1).

### 3.3. Investigation of the Colloid Chemical Stability of Bovine Serum Albumin-Stabilized MnFe_2_O_4_-PB Nanoparticles in Different Solvents

The contrast agent must be redispersed in sterile water, dextrose or physiological saline solution before use. Ferrite nanoparticles stabilized with BSA disperse very quickly in aqueous media, which is an important consideration for their field of application (Figure 10A). Another important requirement is that the colloidal system remains stable for a long time, and some stability tests were performed. We used DLS measurements to track the stability of the water-based colloidal systems over time and monitored changes in particle size. DLS measurements were performed at predefined time points up to 6 h after redispersion. No significant shift in the particle-size distribution was observed during this observation period (Figure 10B). No significant change was observed in the mean and standard deviation values after six hours (Figure 10C). Together, these observations are consistent with effective BSA-associated stabilization of the nanoparticle dispersion during the 6 h observation period. The zeta potentials were measured, initially at −22.2 ± 0.7 mV of the MnFe_2_O_4_-PB/BSA, which remained unchanged after six hours, when it was measured at −22.0 ± 0.3 mV.

When using MRI contrast agents, different solvents are recommended to ensure the appropriate concentration, namely physiological saline solution (0.9 wt%) and dextrose solution (10 wt%). In order to examine how these two solvents affect the colloidal stability of our contrast agent, we performed additional DLS measurements. The mean particle diameters of the MnFe_2_O_4_-PB/BSA were changed in the range 165 ± 46 nm–178 ± 65 nm (in physiological saline solution) and 174 ± 41 nm–201 ± 59 nm (in dextrose solution) based on the intensity-weighted size distribution (Figure 11A). No significant, trend-like change in size distributions was observed for any of the solvents after six hours of measurements. The same conclusion can be drawn with regard to hydrodynamic diameters and polydispersity values (Figure 11B). Based on the above, it can be concluded that, in addition to sterile water for injection, physiological saline solution and dextrose solution can also be used to dissolve the two ferrite-based lyophilized contrast agents.

### 3.4. Mapping Interactions Between Magnetic Nanoparticles and BSA Using Molecular Simulation Methods

BSA-coated nanoparticles form a stable colloidal system in an aqueous medium, as confirmed by DLS studies (Figure 10). The excellent colloidal stability indicates that a strong interaction has developed between BSA and magnetic nanoparticles, making BSA an effective encapsulant for producing lyophilized, redispersible MRI contrast formulations with improved colloidal stability and biocompatibility. Molecular simulation modeling was performed to investigate the interactions between the surface functional groups identified by XPS measurements and BSA.

A 100 ns MD simulation was performed for BSA in aqueous solution and BSA in the presence of three ferrite nanoparticles (MnFe_2_O_4_) (a radius of 25 Å). Throughout the entire simulation period, all three nanoparticles remained in close contact with the protein surface, indicating stable protein–nanoparticle interactions under the applied conditions. The final configurations obtained after 100 ns for both the isolated protein and the protein–nanoparticle complex are shown in Figure 12D. Structural differences between the initial and final states of each system are illustrated in Figure 12C,D, highlighting the conformational changes induced during the simulation.

Root mean square deviation (RMSD) is a widely used metric to assess protein stability, as it quantifies the average structural deviation over time relative to a reference conformation, providing insight into conformational changes and equilibrium behavior during simulations. The RMSD of the protein in both simulations, pure BSA in the solution and BSA with ferrite magnetic nanoparticles (MNPs) in the solution, is comparable (Figure 13A). In the simulation without nanoparticles, the protein initially deviates more strongly from its starting structure, but after about 60 ns, its conformation approaches the initial structure more closely than in the nanoparticle-bound system. In contrast, the RMSD of the protein in the presence of the magnetic nanoparticles increases more gradually and reaches a stable plateau after approximately 50 ns. At equilibrium, the RMSD of the protein in complex with MNPs is slightly higher than that of the pure protein. These results suggest that the MNPs exert a stabilizing influence on the protein structure, reducing abrupt conformational fluctuations while maintaining overall structural integrity. The RMSD of the individual nanoparticles relative to the protein is shown in Figure 13B (the arbitrary numbering of the nanoparticles is defined in Figure 1). All three nanoparticles exhibit notable deviations from their initial positions, which is expected given that the initial configurations were manually placed. However, after approximately 50 ns of simulation, the nanoparticle positions stabilize, indicating equilibration. Figure 13B also shows that magnetic nanoparticle 2 (MNP 2) undergoes the largest shift from its initial structure compared to magnetic nanoparticle 3 (MNP 3) and, to a lesser extent, magnetic nanoparticle 1 (MNP 1).

The minimum distance between each magnetic nanoparticle and the protein remains relatively stable throughout the 100 ns simulation (Figure 13C), fluctuating between 2.0 and 2.2 Å, indicating persistent close contacts. Analysis of the number of contacts, defined as protein and nanoparticle atoms within 5 Å, reveals that for MNP 1 and MNP 2 (as labeled in Figure 1), the number of contacts decreases from ~300 to ~150 within the first nanosecond and then remains relatively constant for the remainder of the simulation (Figure 13D), suggesting stable interactions.

In contrast, MNP 3 exhibits a significantly higher number of contacts, reaching about 400, which stabilize after approximately 20 ns. During the initial 20 ns, the number of contacts for MNP 3 fluctuates considerably, reflecting initial rearrangements. The higher and more stable contact number for MNP 3 indicates a stronger interaction with the protein compared to MNP 1 and MNP 2, consistent with its lower RMSD observed in Figure 13B.

For further insights, the interactions between individual MNPs and protein amino acid residues were further analyzed. For each frame of the molecular dynamics simulation (50 ps per frame), the minimum distance between each nanoparticle and all protein amino acid residues was calculated. This distance was defined as the shortest distance between any atom of the analyzed MNP and any atom of the corresponding amino acid residue. Amino acid residues exhibiting a minimum distance to the nanoparticle of less than 3 Å (0.3 nm) for at least 5% (5 ns) of the total 100 ns MD simulation time were selected for further analysis (Table 2).

The minimum distances between the magnetic nanoparticles and the amino acid residues identified as contacts (Table 2) during the MD simulation are shown in Figure 14. For manganese ferrite MNP 1, the most persistent contact is with Glu293 in chain B, which maintains a stable distance of approximately 2 Å throughout the entire MD simulation. Lys273 in chain B also exhibits a stable, though slightly more fluctuating, distance of 2–3 Å over the full simulation time (Figure 14A). For MNP 2, the most persistent contact is observed with Glu92 in chain B, maintaining a stable distance of around 2 Å throughout the simulation. The interaction between MNP 2 and Lys51 in chain A is also relatively stable at approximately 2 Å; however, this contact is lost after 42 ns of the MD simulation. This residue is partially replaced by Lys76 in chain A for a short period between 57 and 69 ns of the simulation (Figure 14B).

The most persistent contacts for manganese ferrite MNP 3 are with Gln319 and Glu320 in chain B, both maintaining a stable distance of approximately 2.2 Å throughout the entire MD simulation (Figure 14C,D). The interactions with Ala321 and Lys322 are also relatively stable, with distances of around 3 Å during the simulation. The persistently short distance between the nonpolar residue Ala321 and MNP 3 is likely due to strong interactions of the neighboring residues Glu320 and Lys322 with the nanoparticle, rather than a direct interaction between Ala321 and the nanoparticle itself.

Previous analyses indicated a substantially stronger overall interaction between MNP 3 and the protein, which is further supported by the contact analysis. Two additional persistent contacts of MNP 3 (with Lys239 and Lys316 in chain B) are formed after approximately 15 ns of the MD simulation and remain stable at around 2 Å until the end of the simulation (Figure 14C,D). Moreover, another persistent contact with Arg208 in chain B is established after 30 ns and is maintained at approximately 2 Å for the remainder of the simulation.

### 3.5. Testing of the Ferrite-Based Nanoparticles as MRI Contrast Agents

In order to characterize the capabilities of MnFe_2_O_4_-PB/BSA as MRI contrast agents, in vitro relaxivity measurements were conducted. Quantitative images were calculated to determine R1, R2, and R2* relaxation rates of every sample in the diluted concentration rows of the MnFe_2_O_4_-PB nanoparticles. Linear fitting to these data allowed the determination of the r1, r2, and r2* relaxivities of the BSA-coated ferrite nanoparticles (Figure 15).

The longitudinal relaxivities (r1) of the MnFe_2_O_4_-PB/BSA samples were determined to be 0.0026 (mg/mL)^−1^ms^−1^. The transversal relaxivities were measured to be (r2: 1.91 (mg/mL)^−1^ms^−1^; r2*: 4.24 (mg/mL)^−1^ms^−1^). The measured relaxivities indicate that incorporation of BSA preserved the predominantly T2-weighted contrast behavior previously reported for the PB-coated ferrite system [39], while the obtained values remained within the range reported for established SPIO-based contrast agents. The co-formation of magnetite (34.2 wt%) during the PB coating process may contribute additively to the transverse relaxivity of the composite, since magnetite is an established T2-weighted contrast material. This mixed-phase character of the MnFe_2_O_4_-PB material is reflected in the high r_2_ value observed.

Clinically applied superparamagnetic iron oxide formulations and engineered ferrite nanoparticles generally exhibit a predominant effect on transverse relaxation, with substantially higher transverse than longitudinal relaxivity values. Similar to these systems, the MnFe_2_O_4_-PB/BSA nanoparticles demonstrated a strong T_2_-weighted contrast profile, characterized by a low r_1_ value (0.0026 (mg/mL)^−1^ms^−1^) and substantially higher transverse relaxivities (r_2_ = 1.91 (mg/mL)^−1^ms^−1^; r_2_* = 4.24 (mg/mL)^−1^ms^−1^). MRI contrast agents used in clinical practice have higher measured r1 and r2 values; in the case of Endorem, these are r1: 0.0734 (mg/mL)^−1^ms^−1^ (4.1 mM^−1^ s^−1^) and r2: 1.67 (mg/mL)^−1^ms^−1^ (93 mM^−1^ s^−1^). These values for Resovist are r1: 0.0824 (mg/mL)^−1^ms^−1^ (4.6 mM^−1^ s^−1^) and r2: 2.56 (mg/mL)^−1^ms^−1^ (143 mM^−1^ s^−1^) [65]. The r2/r1 ratio was 23 for Endorem and 31 for Resovist [65]. In the case of our MnFe_2_O_4_-based sample, the resulting r_2_/r_1_ ratio (~735) indicates a pronounced preference toward transverse relaxation enhancement. The r_2_/r_1_ ratio is dimensionless and comparable across both unit systems, as the conversion factor cancels. It should be noted that direct numerical comparison between studies remains challenging due to variations in magnetic field strength, pulse sequences, temperature, particle morphology, surface modification, and relaxivity normalization approaches.

### 3.6. Results of the Toxicology Measurement

The cytotoxicity of Prussian blue (PB)-coated manganese ferrite nanoparticles was compared with that of their uncoated counterparts using the Alamar Blue assay. Although the Alamar Blue assay alone measures only general metabolic activity and cell viability, and does not elucidate specific cytotoxicity mechanisms (e.g., ROS generation or inhibition of the Fenton reaction), it effectively illustrates the differences in biocompatibility between the coated and uncoated nanoparticles.

The cytotoxicity of Prussian blue (PB)-coated manganese ferrite nanoparticles was compared with that of their uncoated counterparts using the Alamar Blue assay. The presence of FBS in the culture medium introduces a competitive protein environment that will partially remodel the nanoparticle surface corona during the assay. This condition reflects a physiologically relevant challenge rather than an artifact, and the comparative result between BSA-formulated and BSA+PB-formulated nanoparticles remains internally valid as both were tested under identical conditions.

Although the Alamar Blue assay alone measures only general metabolic activity and cell viability and does not elucidate specific cytotoxicity mechanisms (e.g., ROS generation or inhibition of the Fenton reaction), it effectively illustrates the differences in biocompatibility between the coated and uncoated nanoparticles.

Across the tested concentration range (0.025–1 mg/mL ferrite), all samples exhibited concentration-dependent effects on cell viability (Figure 16).

Mn-containing nanoparticles displayed a concentration-dependent effect on cell viability. Within the BSA-formulated system, the PB-coated formulation showed higher relative metabolic activity than the corresponding uncoated formulation, with values ranging from 52% to 87% and 20% to 34%, respectively, across the tested concentration range. For both formulations, viability decreased progressively with increasing ferrite concentration, reaching its lowest value at 1 mg/mL. These concentration-dependent cell survival data demonstrate that the protective effect of the PB shell remains effective even at higher dosage levels (up to 1 mg/mL). Uncoated particles exhibit toxicity even at the lowest dose (34% viability), whereas PB-coated particles maintain a viability of over 50% even at the highest dose (1 mg/mL).

Within the BSA-formulated nanoparticle system, the PB-coated formulation showed higher relative metabolic activity than the corresponding MnFe_2_O_4_/BSA formulation. The toxicity-reducing effect of the PB coating is understandable given that uncoated ferrite nanoparticles exert their cytotoxic effect through the leaching of transition metal ions (Fe^3+^, Fe^2+^ and Mn^2+^), which catalyze the production of intracellular reactive oxygen species (ROS) via Fenton and Haber–Weiss reactions [8,9,10,11,12,13,14,15]. The PB layer on the surface of the manganese ferrite nanoparticles (the presence of which was confirmed by both XRD and XPS measurements) acts as a passivating layer that inhibits the leaching of the aforementioned heavy metal ions, thereby suppressing ROS generation processes. Furthermore, as demonstrated by our MD simulations, the surrounding BSA matrix forms a persistent protein shield through multivalent electrostatic interactions (e.g., with Glu293 and Lys273 residues). The observed difference in metabolic activity is consistent with a protective effect of the PB coating within the BSA-formulated system. However, the present experimental design does not allow the independent contribution of BSA to cytocompatibility to be quantified. However, Duan and colleagues reported the protective effect of BSA on a graphene oxide/BSA system [66].

### 3.7. Pilot In Vivo MRI Assessment of MnFe_2_O_4_-PB/BSA

A pilot in vivo MRI experiment was performed in a single female BALB/c mouse to assess the feasibility of detecting hepatic accumulation of MnFe_2_O_4_-PB/BSA after intravenous administration. A formulation containing 1 mg/mL ferrite was administered at a volume of 0.1 mL by tail-vein injection. The animal weighed 28.6 g; the administered dose corresponded to 3.5 mg/kg body weight. Native MRI scans were acquired before administration, followed by repeated imaging immediately after administration and again on the following day.

The rapid reduction in hepatic signal intensity observed 10 min after administration indicates rapid hepatic accumulation of the nanoparticles. Albumin-based surface formulations have been reported to influence protein adsorption and circulation behavior in other nanoparticle systems [67,68]. Therefore, the observed MRI signal changes should not be interpreted as evidence of prolonged circulation. Instead, the data demonstrate rapid hepatic uptake followed by persistence of the hepatic signal change at the following-day measurement.

Maximum intensity projection visualization of the signal change (post-20 min scan subtracted from the native pre-scan) highlights that, apart from the liver, only those areas that have shifted between the two images show a decrease in signal intensity. The temporal evolution of hepatic signal intensity (Figure 17B) indicates that the hepatic signal change remained detectable at the following-day measurement after the initial rapid uptake.

The rapid hepatic signal change observed in the present pilot experiment is consistent with the known hepatic uptake of appropriately sized magnetic nanoparticles by the reticuloendothelial system. These observations support rapid hepatic accumulation rather than prolonged blood circulation.

Because this experiment was performed in a single animal, the observations are considered preliminary and are not intended to establish population-level biodistribution, pharmacokinetic parameters or systemic safety.

## 4. Conclusions

In this study, an independently prepared batch of amine-functionalized MnFe_2_O_4_ nanoparticles was coated with Prussian blue and subsequently formulated with bovine serum albumin. The structural, spectroscopic and magnetic characterization confirmed the expected ferrite/PB composition of the newly prepared material. The BSA formulation enabled rapid redispersion after lyophilization and maintained colloidal stability throughout the 6 h DLS observation period in the tested aqueous media. Molecular dynamics simulations identified persistent close contacts between the nanoparticle surface and charged or polar BSA residues, supporting stable association between the protein and nanoparticle surface. The BSA-formulated PB-coated nanoparticles retained predominantly T2-weighted MRI contrast properties. In vitro metabolic activity measurements showed higher viability for the PB-coated BSA formulation than for the corresponding uncoated BSA formulation; however, this comparison does not independently quantify the contribution of BSA to cytocompatibility. A pilot in vivo MRI experiment demonstrated rapid hepatic accumulation, with a significant hepatic signal change detected within 10 min and persisting at the following-day measurement. Because the in vivo assessment was performed in a single animal, these findings should be considered preliminary and cannot establish population-level biodistribution, pharmacokinetics or systemic safety. Further controlled studies are required to determine the specific contribution of BSA to cytocompatibility and pharmacokinetic behavior and to evaluate the translational potential of the formulation.

## Figures and Tables

**Figure 1:**
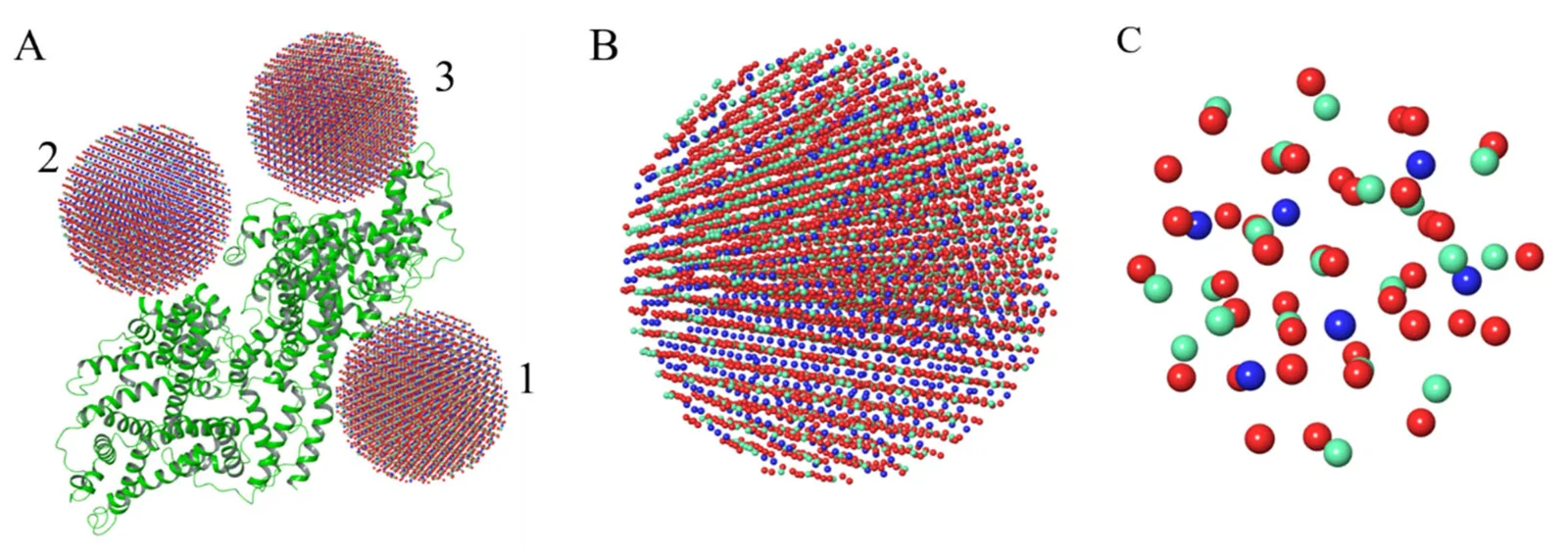
Initial structural model of BSA in complex with three MnFe_2_O_4_ nanoparticles used in the simulations. The numerical labels indicate the arbitrary identifiers assigned to each MnFe_2_O_4_ nanoparticle throughout the study (**A**). Structural model of the nanoparticle used in the simulations (**B**). Simplified MnFe_2_O_4_ model employed for force field parameterization. Color scheme: oxygen (red), manganese (green), and iron (blue) (**C**).

**Figure 2:**
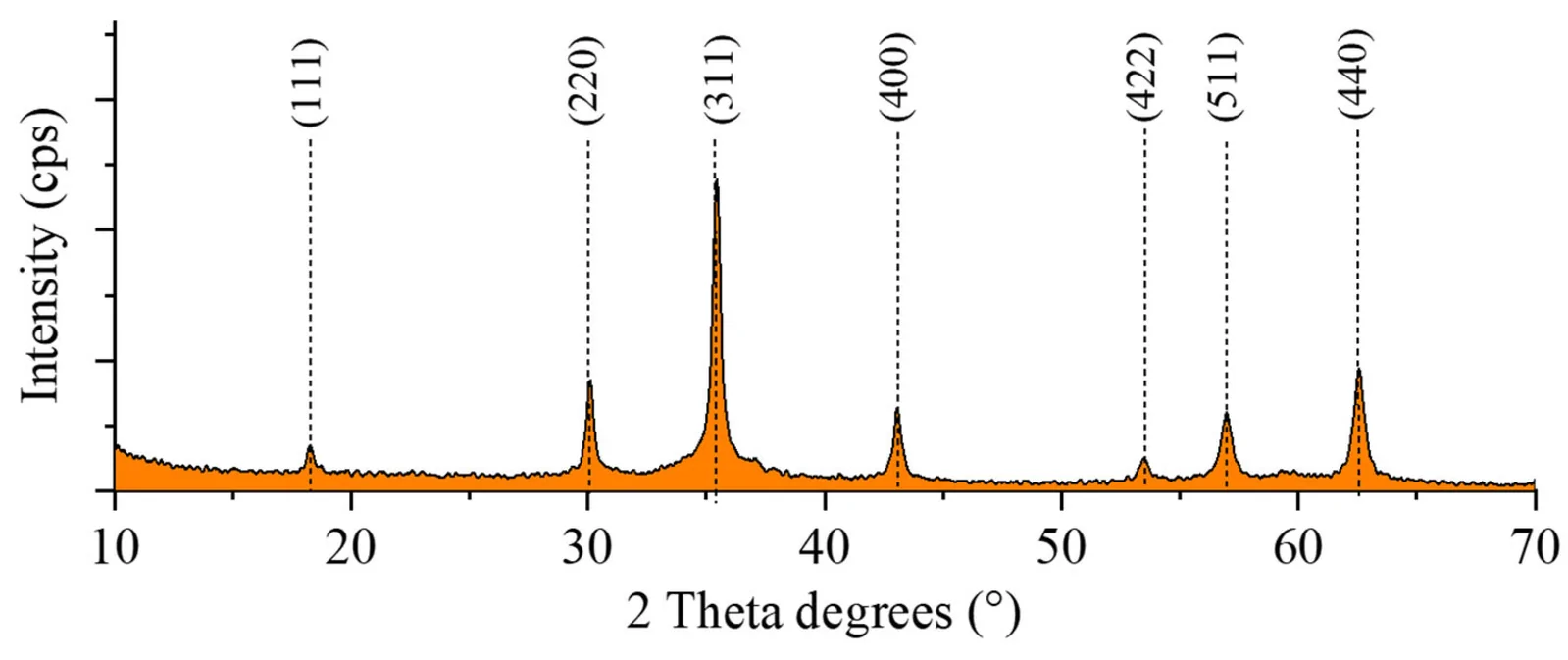
X-ray diffractogram of the MnFe_2_O_4_ nanoparticles [39].

**Figure 3:**
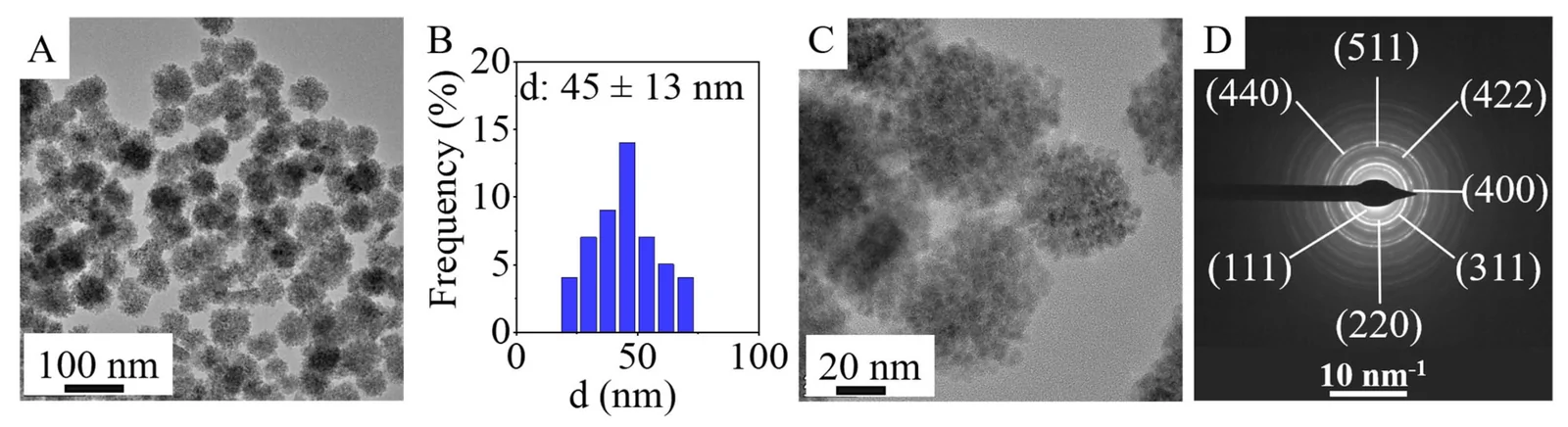
TEM picture (**A**), size distribution histogram (**B**), HRTEM (**C**) and SAED (**D**) picture of the MnFe_2_O_4_ nanoparticles.

**Figure 4:**
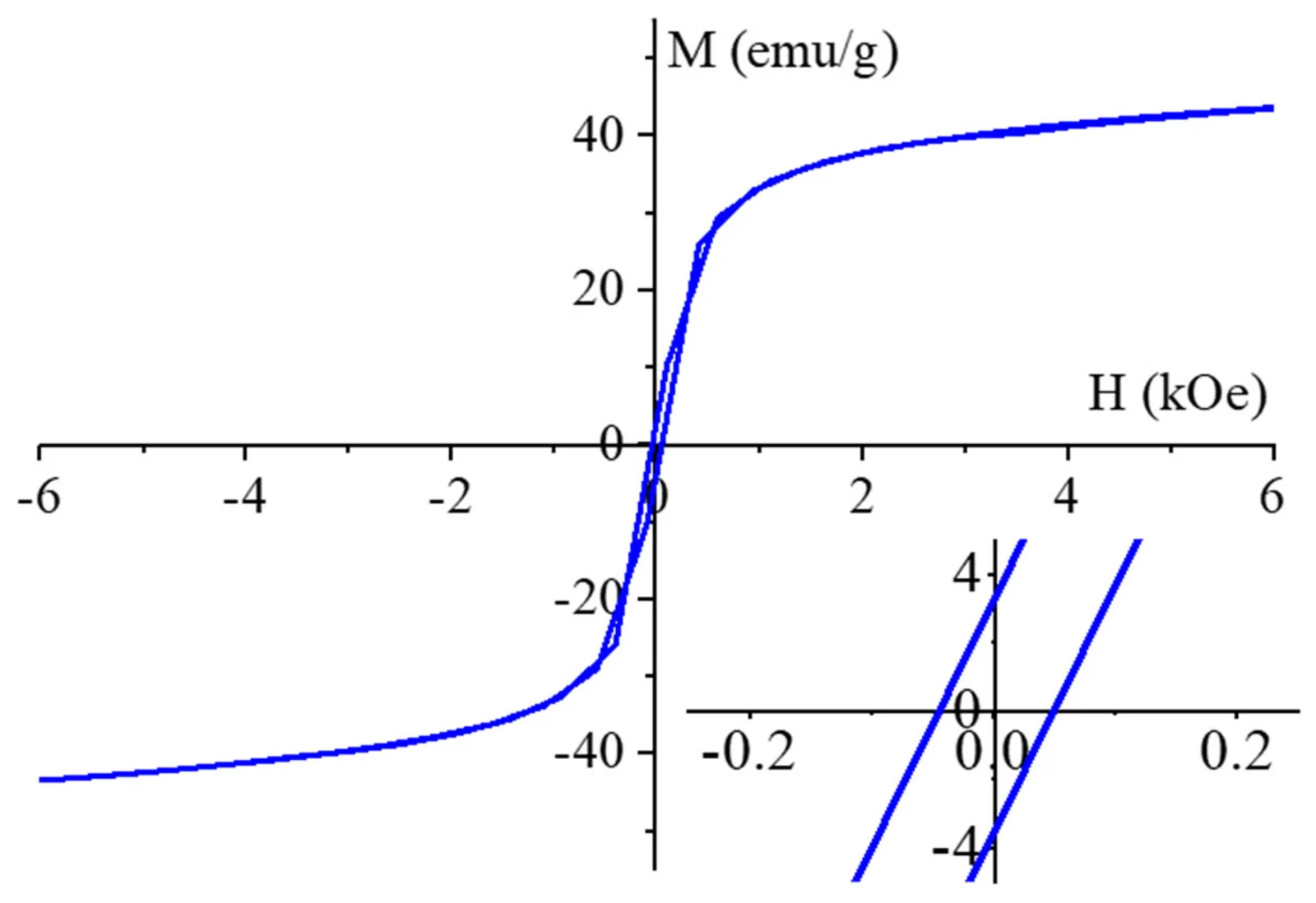
VSM curve of the amine-functionalized MnFe_2_O_4_.

**Figure 5:**
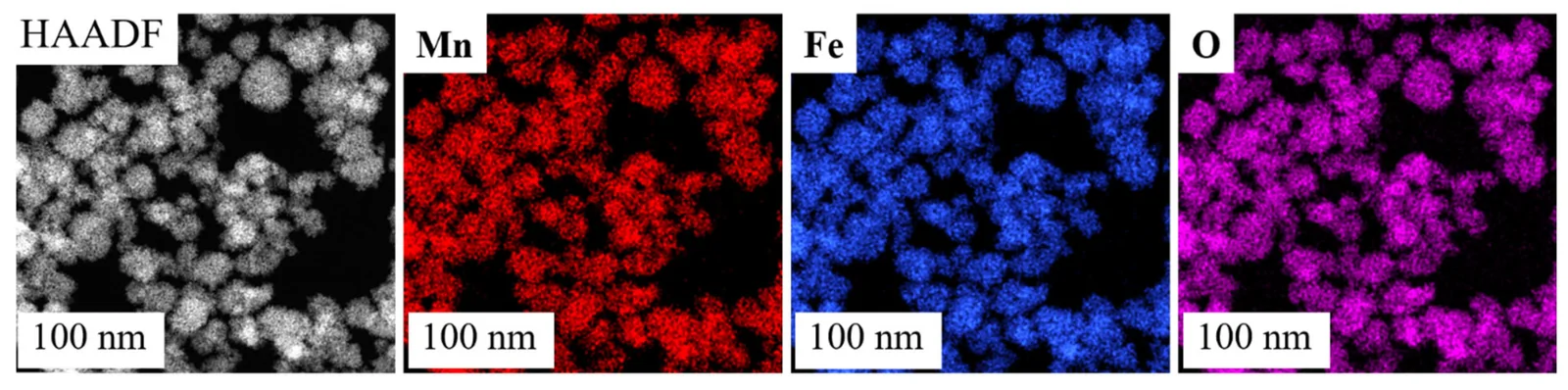
HAADF picture and element maps of the MnFe_2_O_4_-PB nanoparticles.

**Figure 6:**
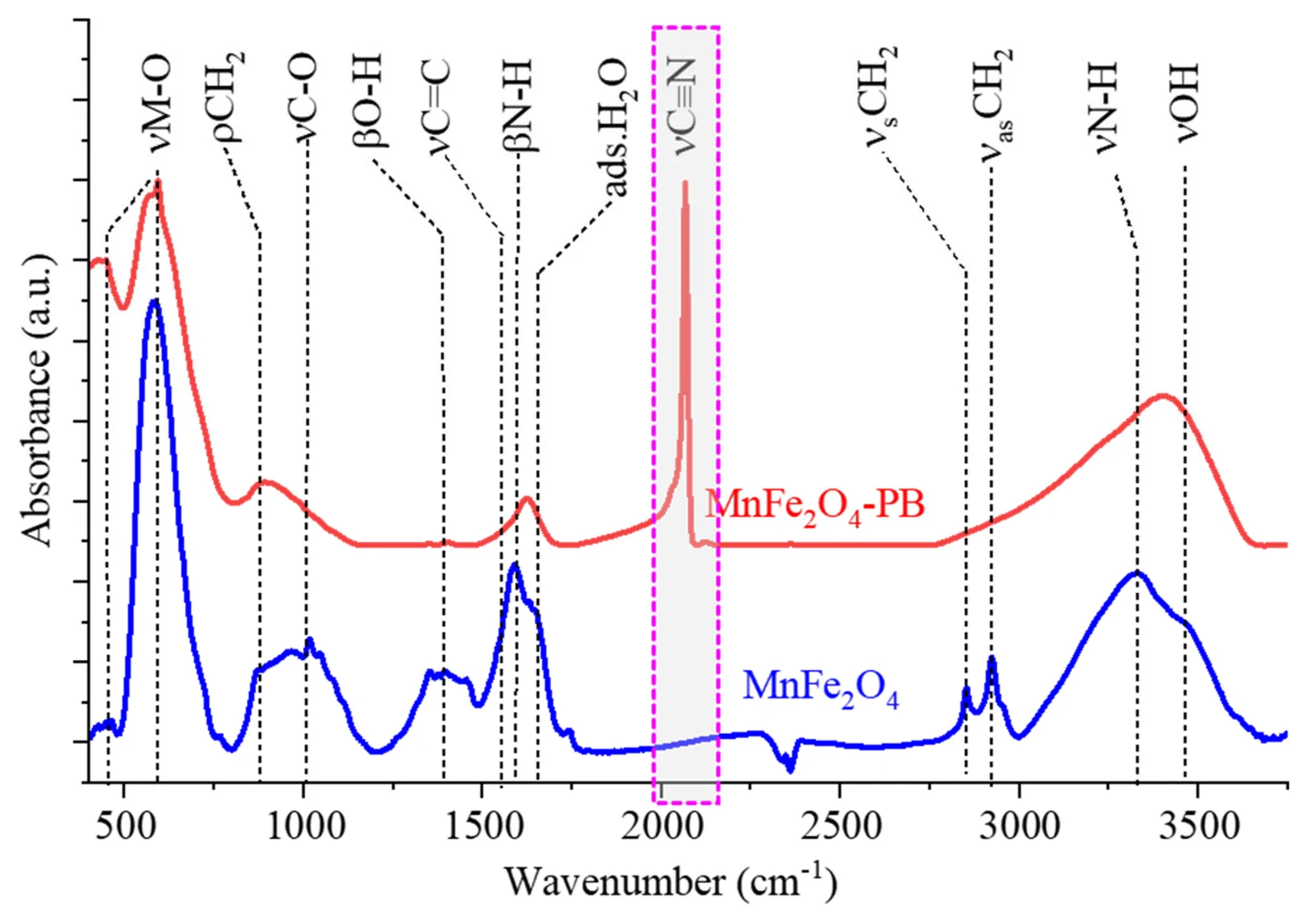
FTIR spectra of the Prussian blue (PB)-coated and the non-treated MnFe_2_O_4_ nanoparticles.

**Figure 7:**
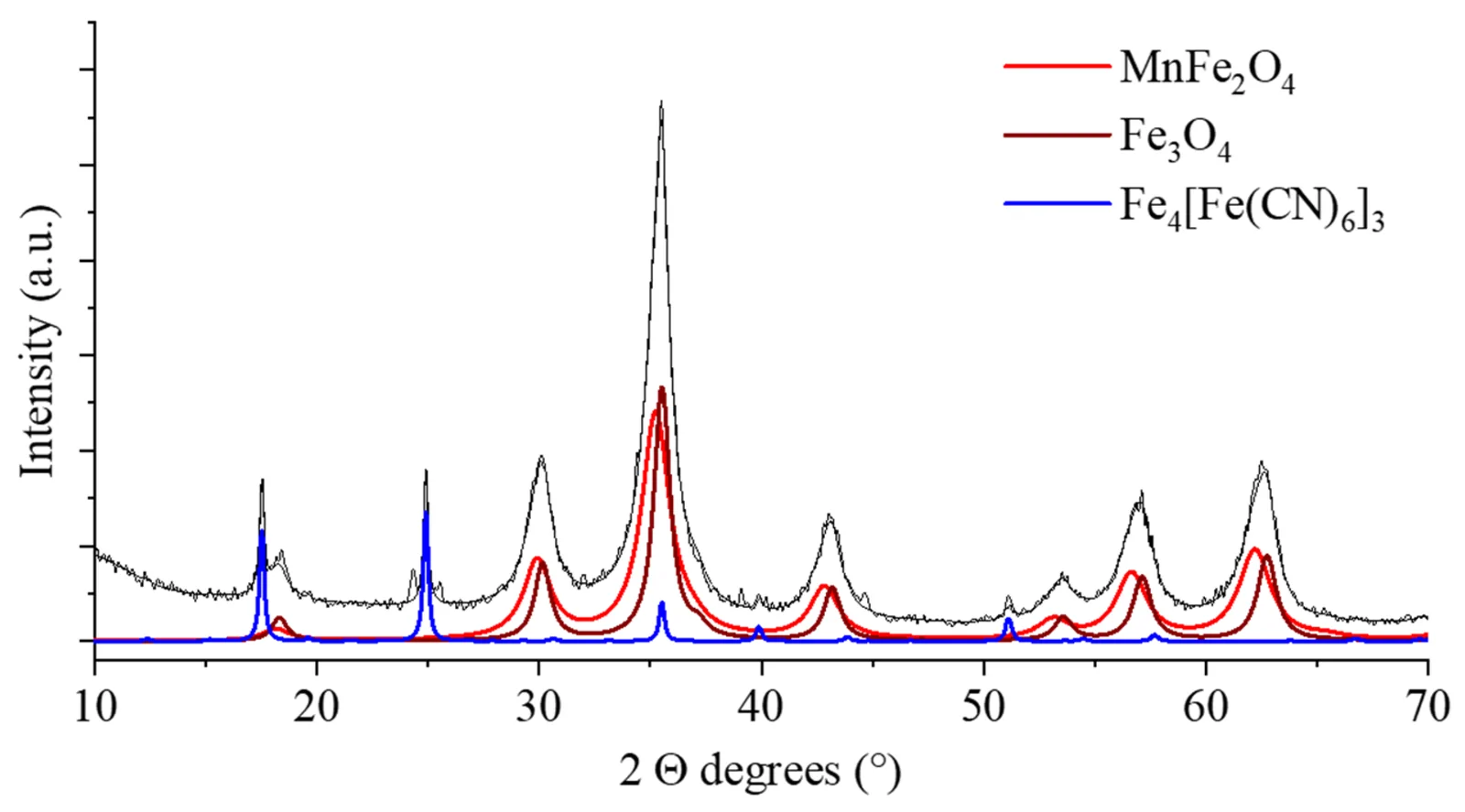
X-ray diffractogram of the MnFe_2_O_4_-PB nanoparticles.

**Figure 8:**
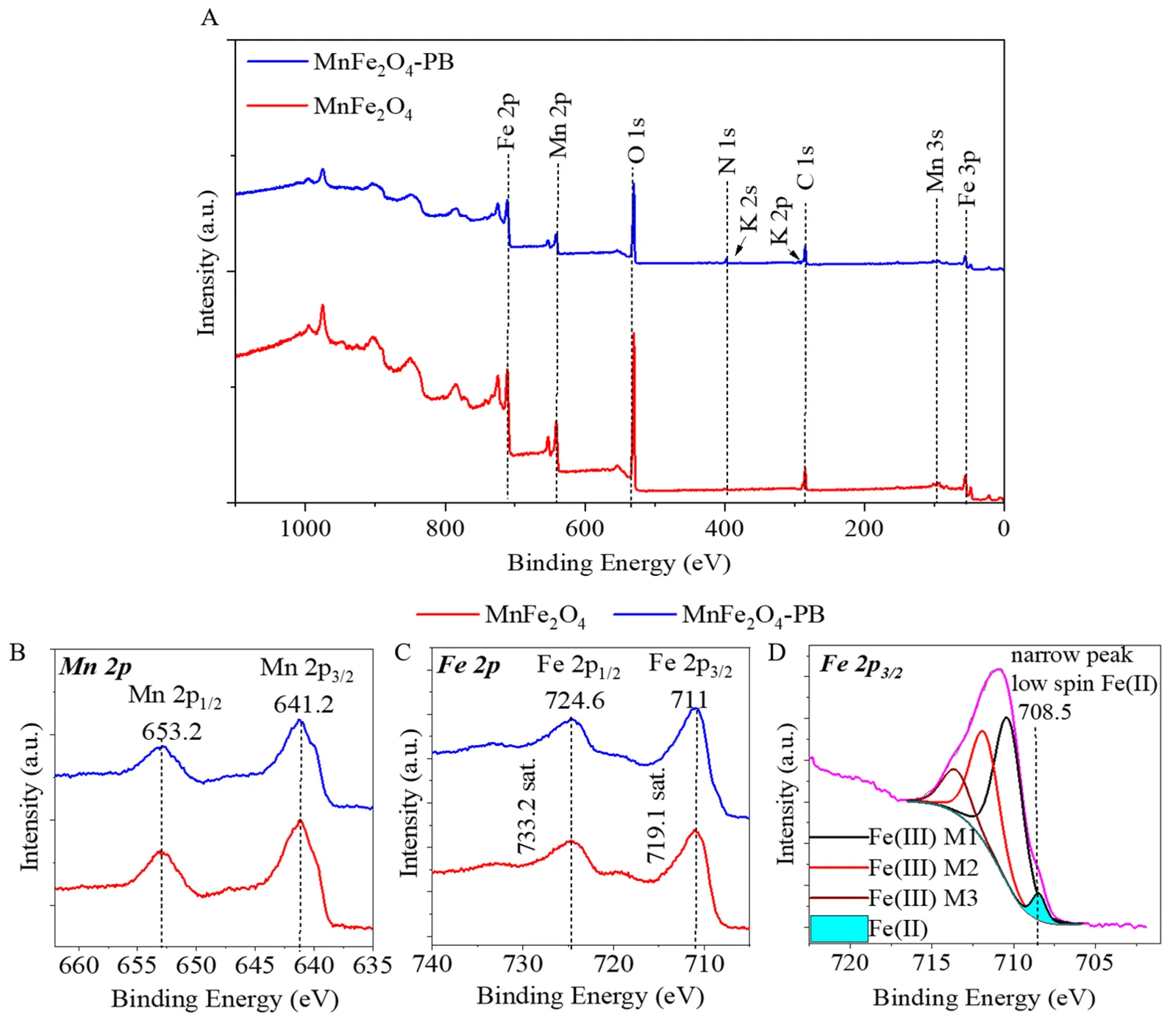
XPS results: survey spectra of the Prussian blue-free and the PB-coated MnFe_2_O_4_ (**A**). Mn 2p bands of the divalent Mn ions (Mn^2+^) in the MnFe_2_O_4_ spinel (**B**). Fe 2p peaks indicate the form of trivalent iron (Fe^3+^) (**C**). The deconvoluted Fe 2p_3/2_ band of the Prussian blue-coated MnFe_2_O_4_ with the peaks of Fe(III) multiplet and Fe(II) (**D**).

**Figure 9:**
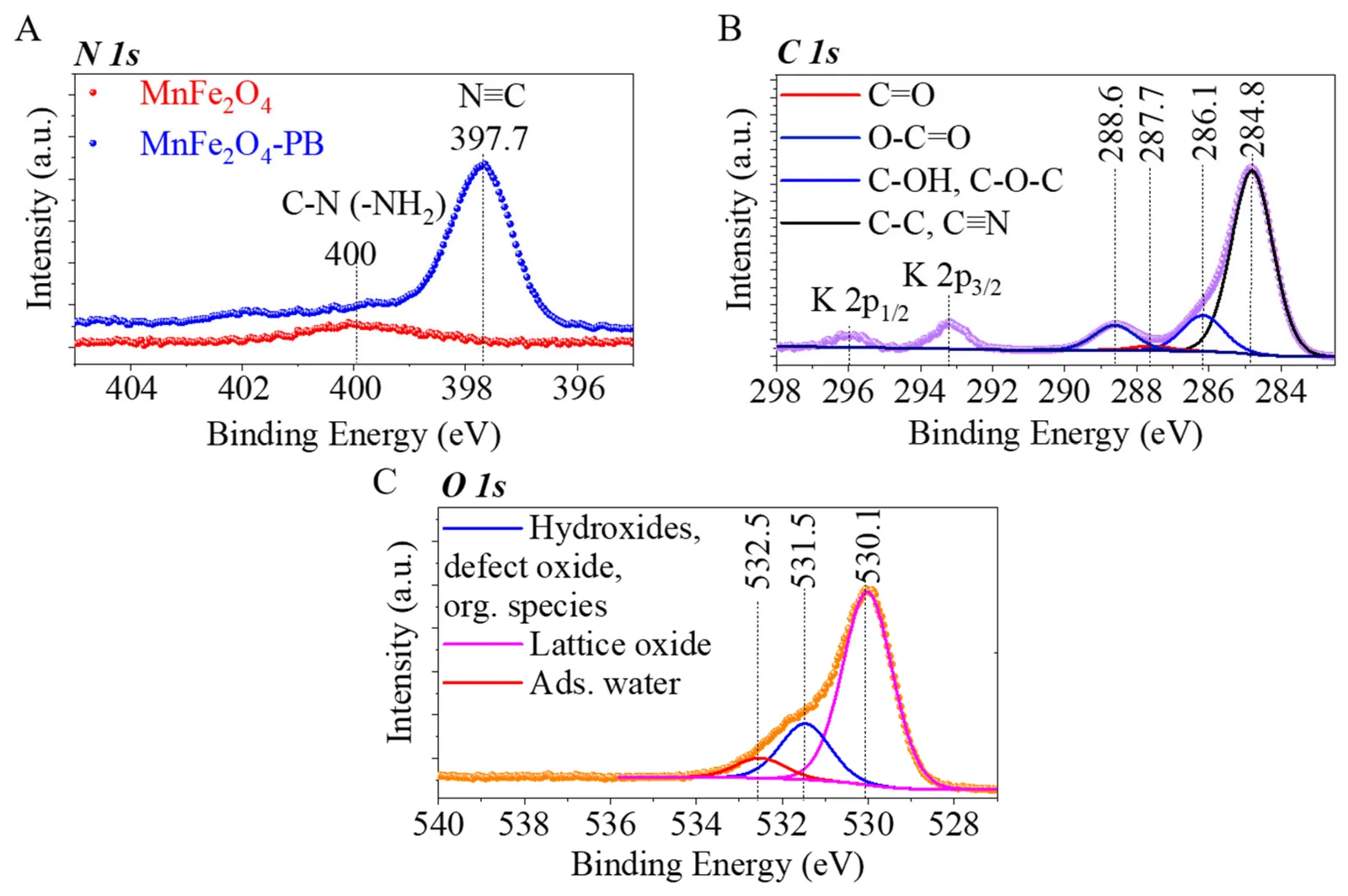
XPS spectra of the N 1s band origin from the amine functional groups (C-N) and N≡C bonds in the Fe^3+^[Fe^3+^Fe^2+^(CN)_6_]_3_ (**A**), C 1s band of the adsorbed organic molecules and Prussian blue in PB-MnFe_2_O_4_ (**B**), and O1 s band of the lattice oxide and functional groups in the pristine and Prussian blue-coated MnFe_2_O_4_ (**C**).

**Figure 10:**
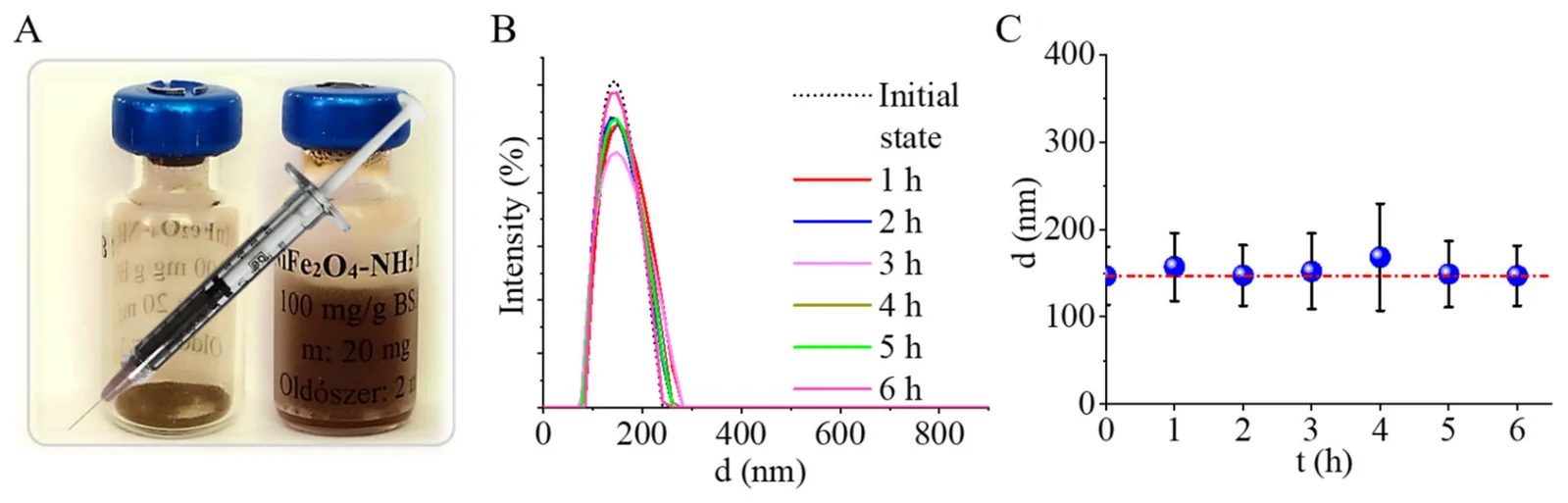
Lyophilized powder of BSA-supported MnFe_2_O_4_-PB contrast agent for injection, and its stable colloid (**A**), size distribution histogram (**B**), and average particle diameters with standard deviations of MnFe_2_O_4_-PB/BSA (**C**) sample stability in time.

**Figure 11:**
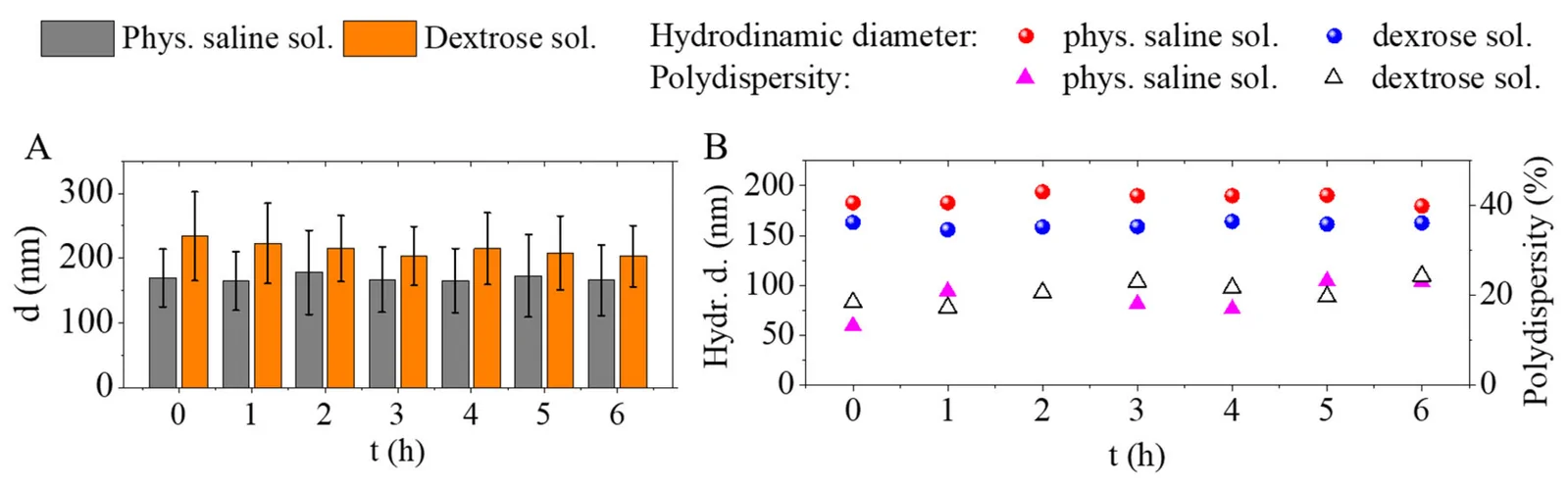
Intensity-weighted size distribution (**A**) and hydrodynamic diameter and polydispersity (**B**) vs. time in different solvents in case of MnFe_2_O_4_-PB/BSA.

**Figure 12:**
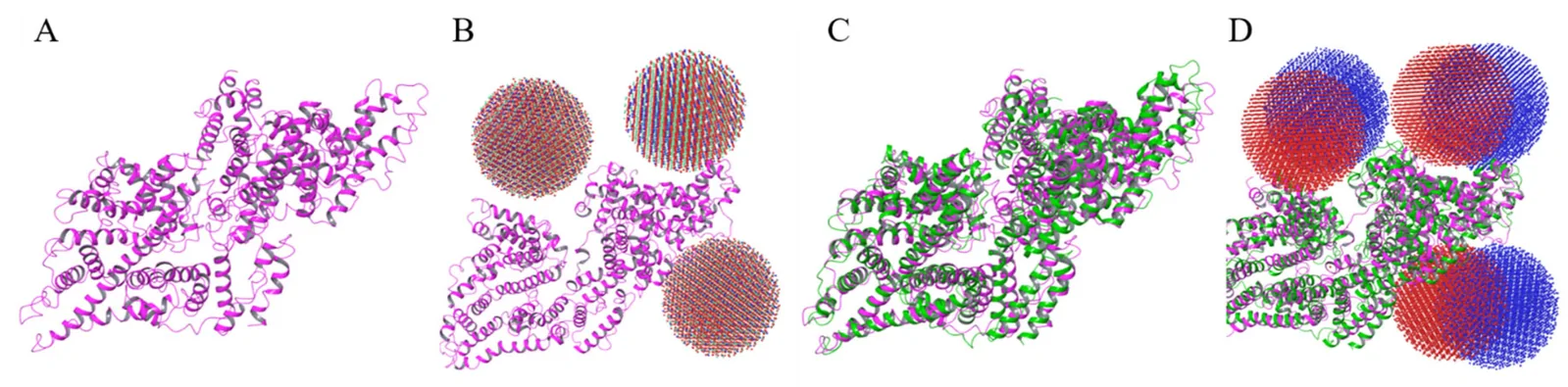
Structures obtained after 100 ns of simulation: BSA in aqueous solution (**A**) and BSA in complex with three manganese ferrite nanoparticles (**B**). Comparison of the initial and final structures after 100 ns of simulation: BSA alone (**C**); BSA in complex with three ferrite nanoparticles (**D**). Initial structures are shown with protein in green and ferrite in red, while final structures are shown with protein in magenta and the ferrite in blue.

**Figure 13:**
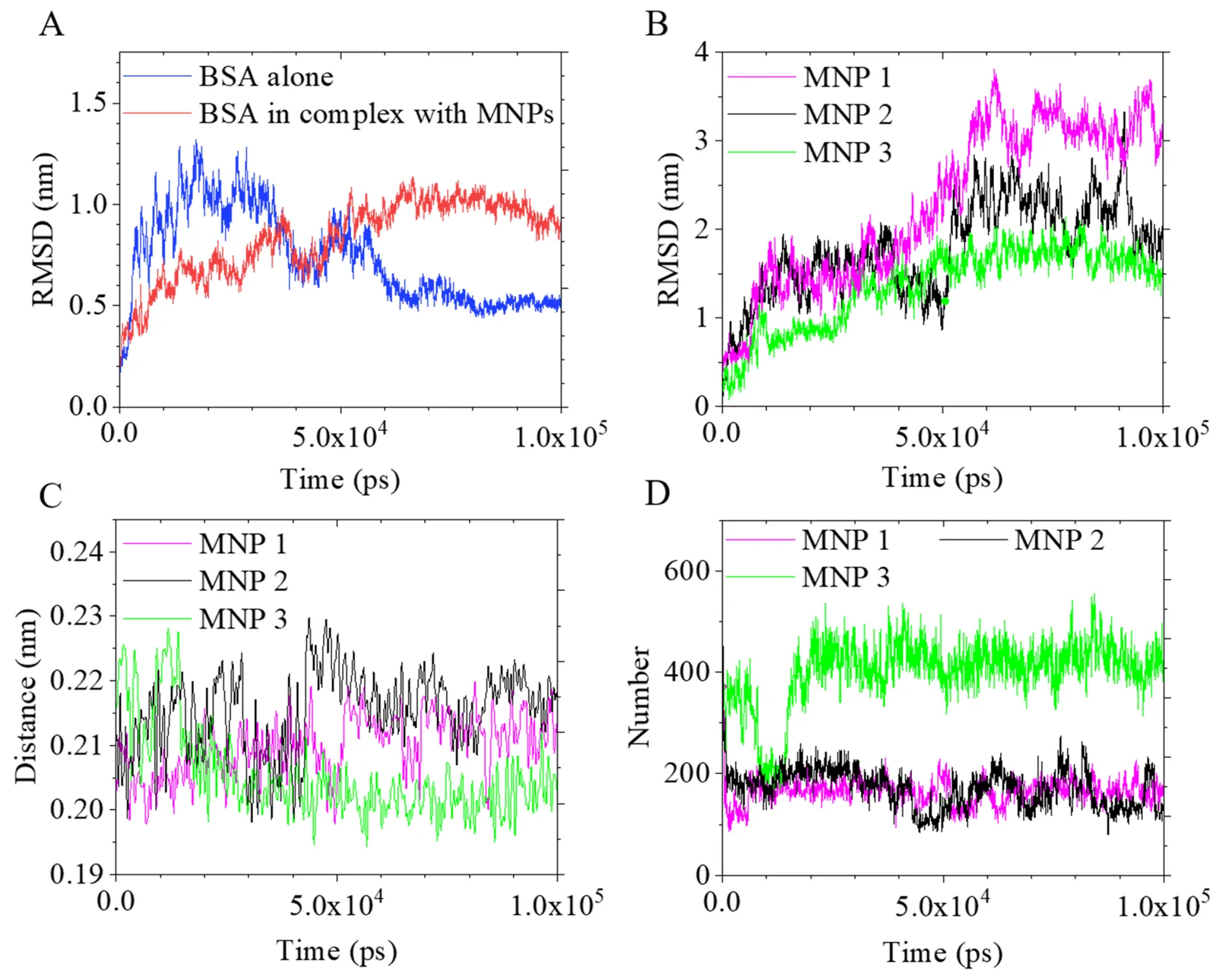
RMSD of protein over the course of the simulation (**A**) and RMSD of the individual MNPs (magnetic nanoparticles) relative to the protein during the simulation (**B**). Minimum distance between each MNP and the protein during the simulation (**C**) and the number of contacts (atoms within 0.5 nm) between each MNP and the protein during the simulation (**D**).

**Figure 14:**
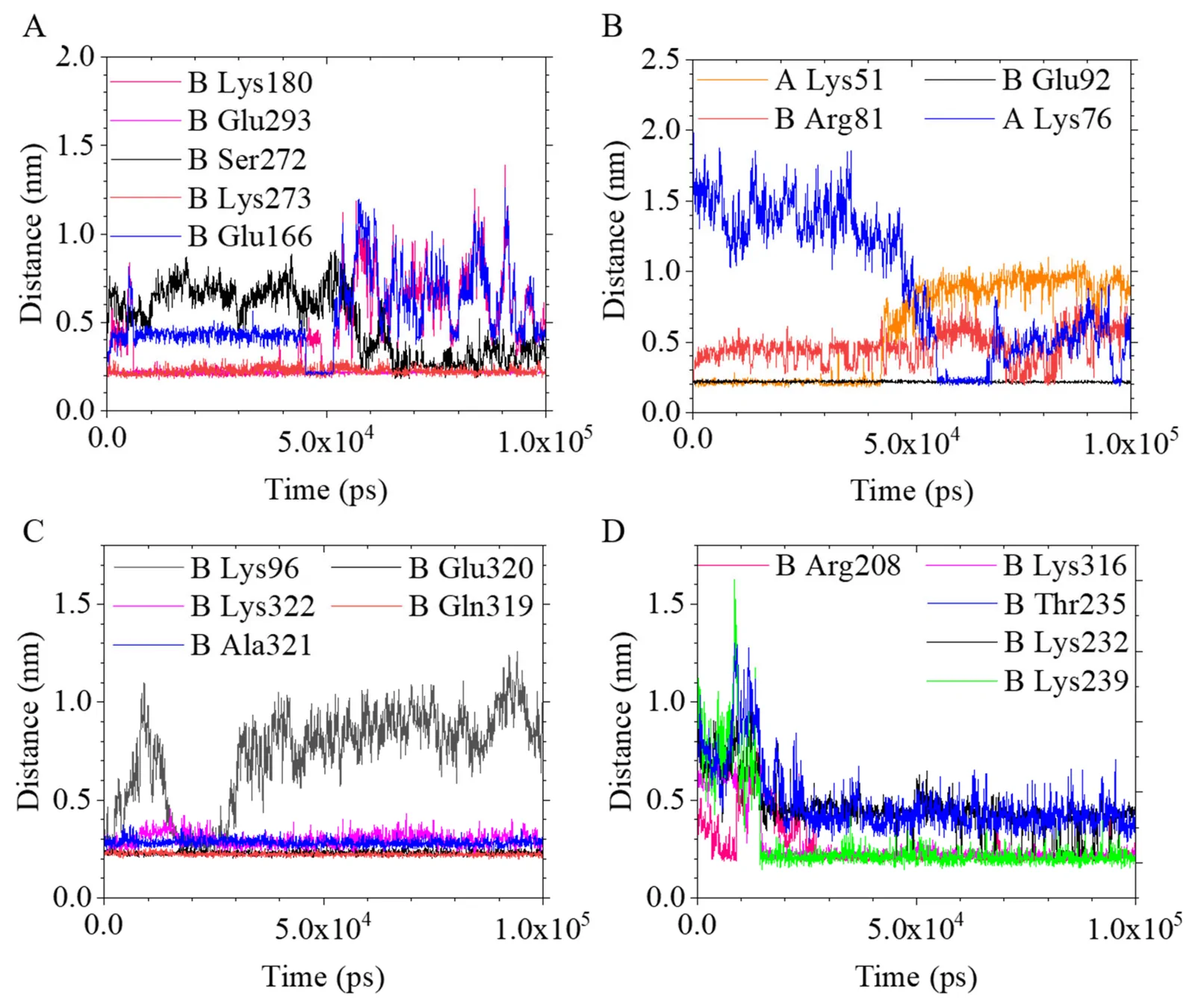
Minimum distance between individual magnetic nanoparticles and selected protein amino acid residues during the MD simulation. Contacts between MNP 1 (**A**), MNP 2 (**B**), and MNP 3 (**C**,**D**) and the amino acid residues in the BSA protein chain.

**Figure 15:**
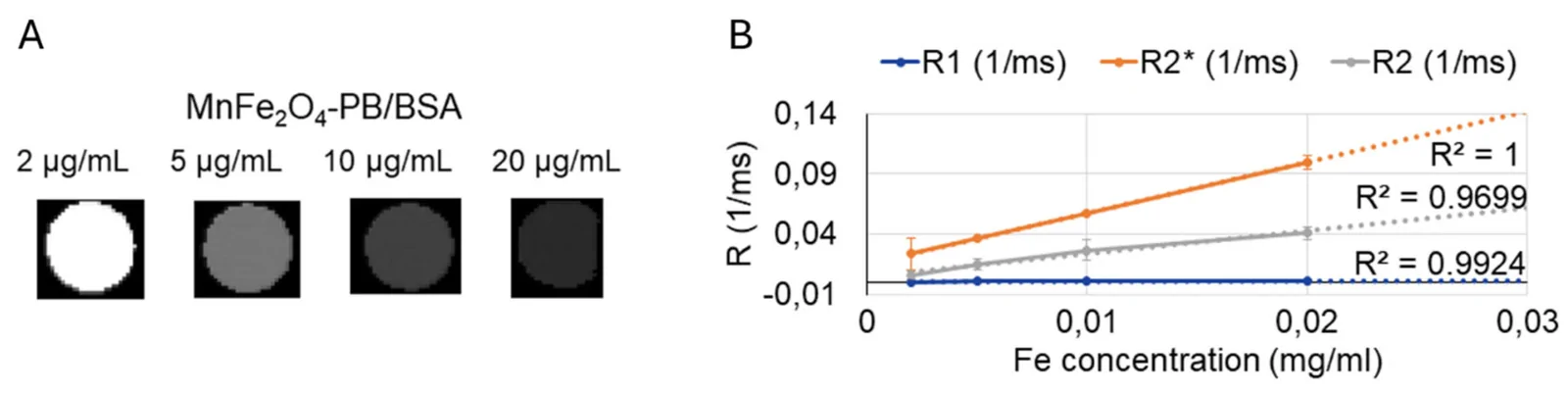
Results of the relaxivity measurements. T2 maps of different concentrations of MnFe_2_O_4_-PB/BSA (**A**) in Eppendorf tubes shown on the same grayscale. The relaxation rates are plotted as a function of the iron content of the samples and linear fitting (dotted lines) to them, the slope of which shows the relaxivity values (**B**). The accuracy of the fits is characterized by the R^2^ values shown in the graph.

**Figure 16:**
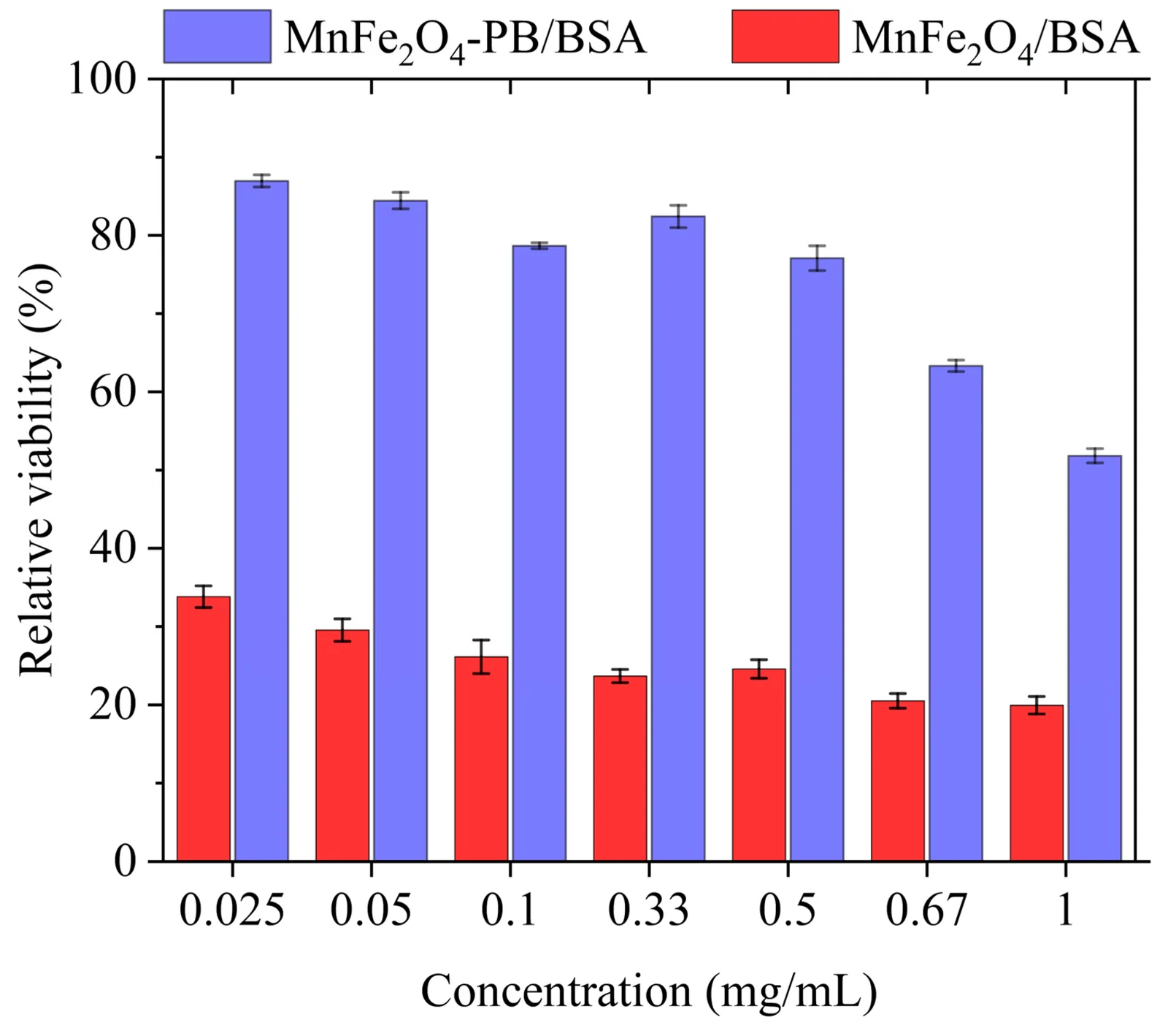
Relative viability of HEK 293T cells exposed to BSA-stabilized MnFe_2_O_4_ nanoparticles, with or without PB coating.

**Figure 17:**
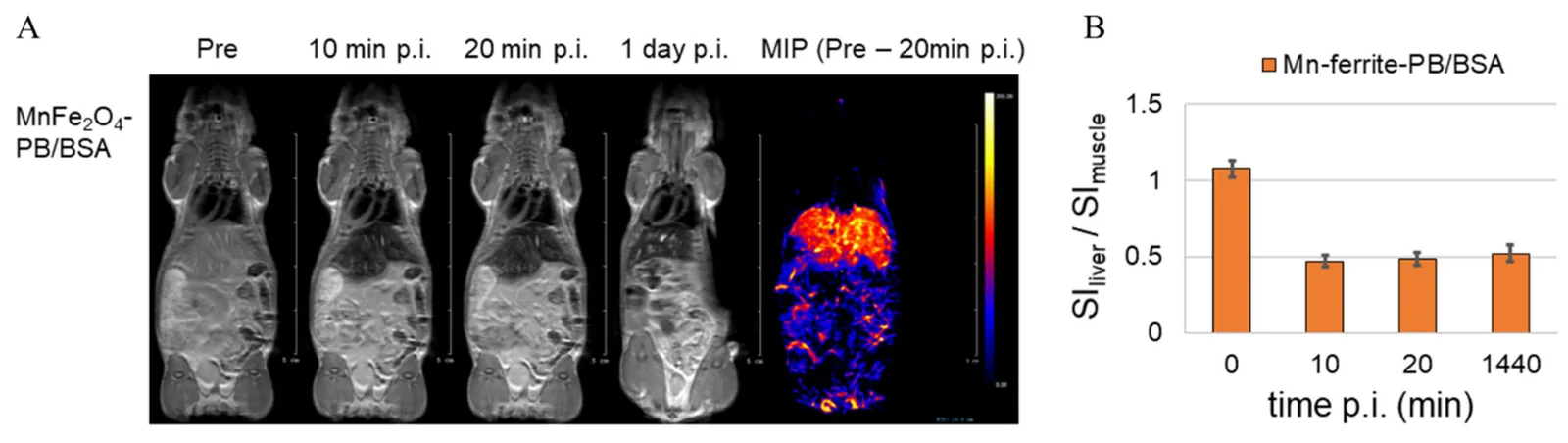
Results of in vivo MRI measurements (**A**). A representative coronal slice of MnFe_2_O_4_-PB/BSA in every timepoint shown on the same grayscale. The last image is a maximum intensity projection (MIP) of the signal intensity change between pre- and 20 min post-injection; mean signal intensities in the liver normalized to the muscle signals are plotted against the time after administration of ferrite-based contrast agent (**B**).

**Table 1:** Surface mole percent (n/n%) of the elements and iron:transition metal ratio in the MnFe_2_O_4_ sample, and in its Prussian blue-modified counterpart.

(n/n)%	C 1s	Fe 3p	Mn 3p	N 1s	O 1s	K 2p	Fe 3p/Mn 3p
**MnFe_2_O_4_**	22	16.6	8.1	0.8	52.5	-	2
**MnFe_2_O_4_-PB**	26.5	15.3	5.8	4.2	47.5	0.7	2.6

**Table 2:** List of amino acid residues in contact (distance < 3 Å) with individual magnetic nanoparticles and the percentage of simulation time during which they remained in contact throughout the MD simulation.

MNP (1)		MNP (2)		MNP (3)	
B Lys180	42%	A Lys51	43%	B Lys96	10%
B Lys273	100%	B Arg81	8%	B Gln319	100%
B Glu293	100%	B Glu92	100%	B Glu320	100%
B Glu166	6%	A Lys76	14%	B Ala321	82%
B Ser272	22%			B Lys322	56%
				B Arg208	81%
				B Lys239	86%
				B Lys316	85%
				B Thr235	14%
				B Lys232	8%

## Data Availability

The datasets used and/or analyzed during the current study are available from the corresponding author on reasonable request.
